# Double-diffusive peristaltic MHD Sisko nanofluid flow through a porous medium in presence of non-linear thermal radiation, heat generation/absorption, and Joule heating

**DOI:** 10.1038/s41598-023-27818-7

**Published:** 2023-01-25

**Authors:** Sayed M. Abo-Dahab, Ramadan A. Mohamed, Abdelmoaty M. Abd-Alla, Mahmoud S. Soliman

**Affiliations:** 1 Mathematics Department, Faculty of Science, South Valley University, Qena, Egypt; 2 Mathematics Department, Faculty of Science, Sohag University, Sohag, Egypt

**Keywords:** Psychology, Materials science, Mathematics and computing, Nanoscience and technology, Physics

## Abstract

This article studied a numerical estimation of the double-diffusive peristaltic flow of a non-Newtonian Sisko nanofluid through a porous medium inside a horizontal symmetric flexible channel under the impact of Joule heating, nonlinear thermal radiation, viscous dissipation, and heat generation/absorption in presence of heat and mass convection, considering effects of the Brownian motion and the thermophoresis coefficients. On the other hand, the long wave approximation was used to transform the nonlinear system of partial differential equations into a nonlinear system of ordinary differential equations which were later solved numerically using the fourth-order Runge–Kutta method with shooting technique using MATLAB package program code. The effects of all physical parameters resulting from this study on the distributions of velocity, temperature, solutal concentration, and nanoparticles volume fraction inside the fluid were studied in addition to a study of the pressure gradients using the 2D and 3D graphs that were made for studying the impact of some parameters on the behavior of the streamlines graphically within the channel with a mention of their physical meaning. Finally, some of the results of this study showed that the effect of Darcy number $Da$ and the magnetic field parameter $M$ is opposite to the effect of the rotation parameter $\Omega$ on the velocity distribution whereas, the two parameters nonlinear thermal radiation $R$ and the ratio temperature ${\theta }_{w}$ works on a decrease in the temperature distribution and an increase in both the solutal concentration distribution, and the nanoparticle's volume fraction. Finally, the impact of the rotation parameter $\Omega$ on the distribution of pressure gradients was positive, but the effect of both Darcy number $Da$ and the magnetic field parameter $M$ on the same distribution was negative. The results obtained have been compared with the previous results obtained that agreement if the new parameters were neglected and indicate the phenomenon's importance in diverse fields.

## Introduction

The peristaltic movement of vital fluids is the natural way in which these fluids move in some organs within the body of a living organism, whether in humans or animals. Knowing that the peristaltic movement that occurs inside the vessels and channels as a result of a force represented in the pressure gradients resulting from the walls of those channels contributes greatly to the transfer of these fluids inside the body of the organism. In the circulatory system, for example, the heart is the pump that works to pump blood into the veins, arteries, and small blood vessels, and thus the movement of blood, in this case, is considered a peristaltic movement under the influence of the so-called systolic pressure and diastolic pressure within the arteries and veins. The peristaltic movement is also clearly visible in the urinary system through the passage of urine through the flexible ureteral canal from the kidney to the urinary bladder. It appears in the male reproductive system through the transfer of semen into the flexible duct of the carrier vessel from the testicle to the outside of the body. Naturally, the peristaltic behavior appears clearly within the elastic channels which widen and narrow based on the movement of the fluid inside them an irregular movement under the influence of strong pressure gradients, this causes the walls of the channel to be formed in the form of transverse mechanical waves consisting of tops and bottoms. Many investigators investigated the peristaltic phenomenon in different disciplines. Accordingly, the scientific meaning of the current problem is the application of peristaltic motion or double-diffusion peristaltic flow on the non-Newtonian Sisko fluid, which contains nanoparticles inside a suitable flexible channel under the influence of some physical factors, and observing what will result from this study. Latha^1^ was the first to study the transmission of fluids by the peristaltic pumping mechanism or the peristaltic wave method. Tanveer et al.^2^ studied the numerical simulation for peristalsis of Sisko nanofluid in a curved channel with double-diffusive convection, the study showed that the effect of the heat Grashof number and the nanoparticles Grashof number on the velocity distribution was positive, in contrast to the effect of the solutal Grashof number on the same distribution. Bibi et al.^3^ investigated the Entropy generation analysis in the peristaltic motion of Sisko material with variable viscosity and thermal conductivity. Bhatt et al.^4^ analyzed the endoscope analysis on peristaltic blood flow of Sisko fluid with titanium magneto-nanoparticles. This study dealt with the process of the medical impact of the medical endoscope and its direct impact on the peristaltic movement of the blood, and this is realistic experimental scientific evidence In the field of medicine on the study of peristaltic motion. Almaneea^5^ dissected a numerical study on the thermal performance of Sisko fluid with hybrid nanostructures. Asghar et al.^6^ demonstrated a mathematical framework for a peristaltic flow analysis of non-Newtonian Sisko fluid in an undulating porous curved channel with heat and mass transfer effects Akram et al.^7^ discussed the Crossbreed impact of double diffusivity convection on peristaltic pumping of magneto Sisko nanofluids in a non-uniform inclined channel. It was found from this study that the effect of the thermophoresis parameter on the temperature distribution was positive, while its effect on the distributions of both nanoparticle volume fraction and solute concentration was negative. Imran^8^ disputed the integrated MHD thermal performance with slip conditions on metachronal propulsion.

Non-Newtonian fluids have many applications, including the manufacture of toothpaste, glues, ink-jet printing, geographical tables, ketchup, the manufacture of pharmaceutical products, and others. On the other hand, you see some biological examples of non-Newtonian fluids in nature, such as emulsions, blood flows, starch suspensions, lubricants, and so on as well. Sisko fluid is one of the most important models of non-Newtonian fluids. It is an extension of the force law fluid model proposed in 1958 by Sisko^9^ and in terms of its physical nature, it also combines its properties between Newtonian and non-Newtonian fluids, and this type of fluid can be found easily in the nature because of its many of applications, also grease flow is the most relevant example of Sisko fluid. The peristaltic motion of Sisko fluid in an inclined asymmetric tapered channel with nonlinear radiation, where the effect of the thermal radiation parameter on the temperature distribution was negative, and this is one of the results of the study of this work investigated by Hayat et al.^10^. Ahmed et al.^11^ construed heat and mass transfer effects on the peristaltic flow of Sisko fluid in a curved channel. Iqbal et al.^12^ examined the effects of convection on Sisko fluid with peristalsis in an asymmetric channel. It was observed from this study that the temperature distribution was increasing under the influence of the first heat transfer Biot number ${Bi}_{1}$ and that the same distribution was increasing under the influence of the second heat transfer Biot number ${Bi}_{2}$. Hayat et al.^13^ excogitated a numerical study for MHD peristaltic transport of Sisko nanofluid in a curved channel. Nawaz et al.^14^ elaborated a numerical study for the peristalsis of Sisko nanomaterials with entropy generation. It was found through that study that the temperature distribution was negative under the influence of the ratio temperature parameter and that the nanoparticle's volume fraction distribution was positive under the influence of the same parameter. Imran et al.^15^ explained the exploration of thermal transport for the Sisko fluid model under the peristaltic phenomenon. Maraj and Nadeem^16^ anatomized a theoretical analysis for the peristaltic flow of Sisko nanofluid in a curved channel with compliant walls. Agoor et al.^17^ dissected the peristaltic flow with heat transfer on Sisko fluid in a ciliated artery. Rashed and Ahmed^18^ studied the peristaltic flow of dusty nanofluids in curved channels. Ahmed and Rashed^19^ investigated magnetohydrodynamic dusty hybrid nanofluid peristaltic flow in curved channels.

In general, porous media are the media or bodies that contain a group of voids called holes or openings, or pores and they have many types such as a sponge, sand, and others. When talking about the flow of fluids through porous media, this means that the fluid permeates the spaces in these media during the process of flow. On the other side, the physical nature of the porous media acts as a force that resists or obstructs the movement of the fluid, which leads to a decrease in the velocity of the fluid and also thus a decrease in its movement. Peristaltic flow through a porous medium in a non-uniform channel mechanism of pumping fluids in a tube utilizing a contractile ring around the tube which pushes the fluid forward. When talking about the vital porous media present inside the flexible channels and tubes, such as the stomach, small intestine, and large intestine, for example, it is represented in the semi-permeable membranes located on the inner layer of the intestinal wall, whether it is the small or large intestine. It is also represented in the food inside the stomach when mixed with the gastric juices that digest food. The cell wall that surrounds the cell inside the body of the organism can be considered a porous medium because it allows the entry of food into the cell from the blood and allows the exit of waste products from the same cell into the blood as well. Abd-Alla et al.^20^ studied peristaltic flow in cylindrical tubes with an endoscope subjected to the effect of rotation and magnetic field. Abd-Alla et al.^21^ investigated the effects of an endoscope and rotation on peristaltic flow in a tube with a long wavelength. Abd-Alla et al ^22^ analyzed magnetic field and gravity effects on the peristaltic transport of a Jeffrey fluid in an asymmetric channel. Abd-Alla et al. ^23^ examined the effect of heat and mass transfer and rotation on peristaltic flow through a porous medium with compliant walls. Mekheimer and Abd-Elmaboud^24^ examined peristaltic flow through a porous medium in an annulus. Mahmoud et al.^25^ anatomized the effect of a porous medium and magnetic field on the peristaltic transport of a Jeffrey fluid. Tariq and Khan^26^ construed the peristaltic flow of a dusty electrically conducting fluid through a porous medium in an endoscope. Abd-Alla and Abo-Dahab ^27^ illustrated the effect of rotation on the peristaltic flow of fluid in a symmetric channel through a porous medium with a magnetic field. Abd-Alla and Abo-Dahab^28^ elaborated on the magnetic field and rotation effects on the peristaltic transport of a Jeffrey fluid in an asymmetric channel. Abd-Alla et al.^29^ illustrated the peristaltic flow of a Jeffrey fluid under the effect of the radially varying magnetic field in a tube with an endoscope.

In recent years, there has been a great leap that was the reason for the tremendous progress in the applications of nanofluids, which made researchers very interested in studying the properties of nanofluids, which contributed greatly to solving the problems industries of both engineering, chemical and nuclear in addition, the study of the properties of thermal or electrical nanofluids played an important role in solving engineering problems such as solar energy collection, heat exchangers and cooling of electric motors. On the other hand, Choi^30^ is considered the first to study nanofluids, and the term nanofluids is a new class of basic fluids such as oil, water, bio-fluids, and ethylene contains ultrafine metal particles whose size is measured in nanometers. These particles have a very high thermal and electrical conductivity. Ahmed et al.^31^ explained the effects of nonlinear thermal radiation, convective boundary conditions, and heat generation/absorption on magnetohydrodynamic Maxwell nanofluid flow over a stretching surface through a porous medium. Mohamed et al.^32^ analyzed the nonlinear thermal radiation and heat generation/absorption effect on MHD Jeffrey nanofluid flow over a stretching sheet through a porous medium. The influences of thermal radiation and slip conditions on MHD Casson nanofluid flow over a stretching surface through a porous medium was studied by Mohamed et al.^33^. Bouslimi et al.^34^ dissected MHD Williamson nanofluid flow over a stretching sheet through a porous medium under the effects of Joule heating, nonlinear thermal radiation, heat generation/absorption, and chemical reaction. Mohamed et al.^35^ analyzed the thermal radiation and MHD effects on the free convective flow of a polar fluid through a porous medium in the presence of internal heat generation and chemical reaction. The MHD three-dimensional flow of a couple stress nanofluids over a stretching sheet through a porous medium in presence of heat generation/absorption and nonlinear thermal radiation was discussed by Mohamed et al.^36^. Abd-Alla et al.^37^ studied the effects of rotation and initial stress on peristaltic transport of fourth-grade fluid with heat transfer and induced magnetic field. The study shows that the velocity distribution decreases with an increase in the rotation coefficient, while the velocity distribution increases with an increase in the heat generation and absorption coefficient. Abd-Alla et al.^38^ examined the rotation and initial stress effect on MHD peristaltic flow of reacting radiating fourth-grade nanofluid with viscous dissipation and Joule heating. Akbar^39^ analyzed the peristaltic Sisko nano fluid in an asymmetric channel. Abd-Alla et al.^40^ investigated the effect of rotation on the peristaltic flow of a micropolar fluid through a porous medium with an external magnetic field. El-Dabe et al.^41^ investigated a couple stress of peristaltic motion of Sutter by micropolar nanofluid inside a symmetric channel with a strong magnetic field and Hall currents effect. Alhazmi et al.^42^ studied Thermal convection in nanofluids for peristaltic flow in a nonuniform channel. Abd‑Alla et al.^43^ analyzed the numerical solution for MHD peristaltic transport in an inclined nanofluid symmetric channel with a porous medium. Raja et al.^44^ examined the integrated intelligent computing application for the effectiveness of Au nanoparticles coated over MWCNTs with velocity slip in curved channel peristaltic flow. Hussain et al.^45^ discussed the thermal radiation impact on bioconvection flow of nano-enhanced phase change materials and oxytactic microorganisms inside a vertical wavy porous cavity. Hussain et al.^46^ analyzed the bioconvection of oxytactic microorganisms with nano-encapsulated phase change materials in an omega-shaped porous enclosure. Hussain et al.^47^ illustrated the natural convection of a water-based suspension containing nano-encapsulated phase change material in a porous grooved cavity. Akbar et al. ^48^ studied the exact solutions of an unsteady thermal conductive pressure driven peristaltic transport with temperature-dependent nanofluid viscosity. Maraj et al.^49^ investigated the framing the MHD mixed convective performance of CNTs in rotating vertical channel inspired by thermal deposition. Akram et al.^50^ elaborated the electroosmosis augmented MHD peristaltic transport of SWCNTs suspension in aqueous media, Journal of Thermal Analysis and Calorimetry. Mohamed et al.^51^ construed the effects of nonlinear thermal radiation and heat generation/absorption on magnetohydrodynamic (MHD) Carreau nanofluid flow on a nonlinear stretching surface through a porous medium. Abd-Alla et al.^52^ discussed heat and mass transfer for MHD peristaltic flow in a micropolar nanofluid: mathematical model with thermophysical features.

The main objectives of the current study are to study the double-diffusive peristaltic non-Newtonian Sisko nanofluid flow under the influence of a magnetic field through a suitable porous medium inside a horizontal symmetric flexible channel under the influence of both Joule heating, heat generation/absorption, viscous dissipation and non-linear thermal radiation in the presence of heat and mass convection, considering the effect of Brownian motion with thermophoresis. On the other hand, the system of differential equations governing the process of peristaltic flow within the symmetric horizontal elastic channel is a set of non-linear partial differential equations in two dimensions transformed into a new system of non-linear ordinary differential equations in one dimension after applying the long wavelength approximation with the small Reynolds number. A MATLAB program was used to solve the new system of nonlinear ordinary differential equations using the fourth-order Runge–Kutta numerical method with an imaging technique. Moreover, graphs were made using the MATLAB program showing the study of the effect of each parameter separately on the main distributions, namely axial velocity, temperature, solute concentration, and nanoparticle volume fraction when the values of the rest of the parameters were constant. Also, the physical meanings of the influences of some important physical parameters on the main distributions mentioned above have been clarified. Table 1. shows an important comparison of research studies for a group of researchers in the field of peristaltic motion of some models of non-Newtonian fluids with the current study.

**Table 1:** A comparison between some previous works.

	Almaneea^5^	Asghar et al.^6^	Akram et al.^7^	Hayat et al.^10^	Iqba et al.^12^	Nawaz et al.^14^	Present study
Nanofluid	No	No	Yes	No	No	Yes	Yes
Viscous dissipation	No	No	No	No	No	No	Yes
Double diffusive	No	No	Yes	No	No	No	Yes
Porous medium	No	Yes	No	No	No	No	Yes
Thermal radiation	No	No	No	Yes	No	Yes	Yes
Joule heating	No	No	No	No	No	No	Yes
MHD	Yes	No	No	No	No	Yes	Yes
Rotation	No	No	No	No	No	No	Yes
Heat generation	Yes	No	No	No	No	No	Yes
Heat convection	No	No	No	No	No	No	Yes
Mass convection	No	No	No	No	No	No	Yes

## Mathematical formulation

**Figure 1:**
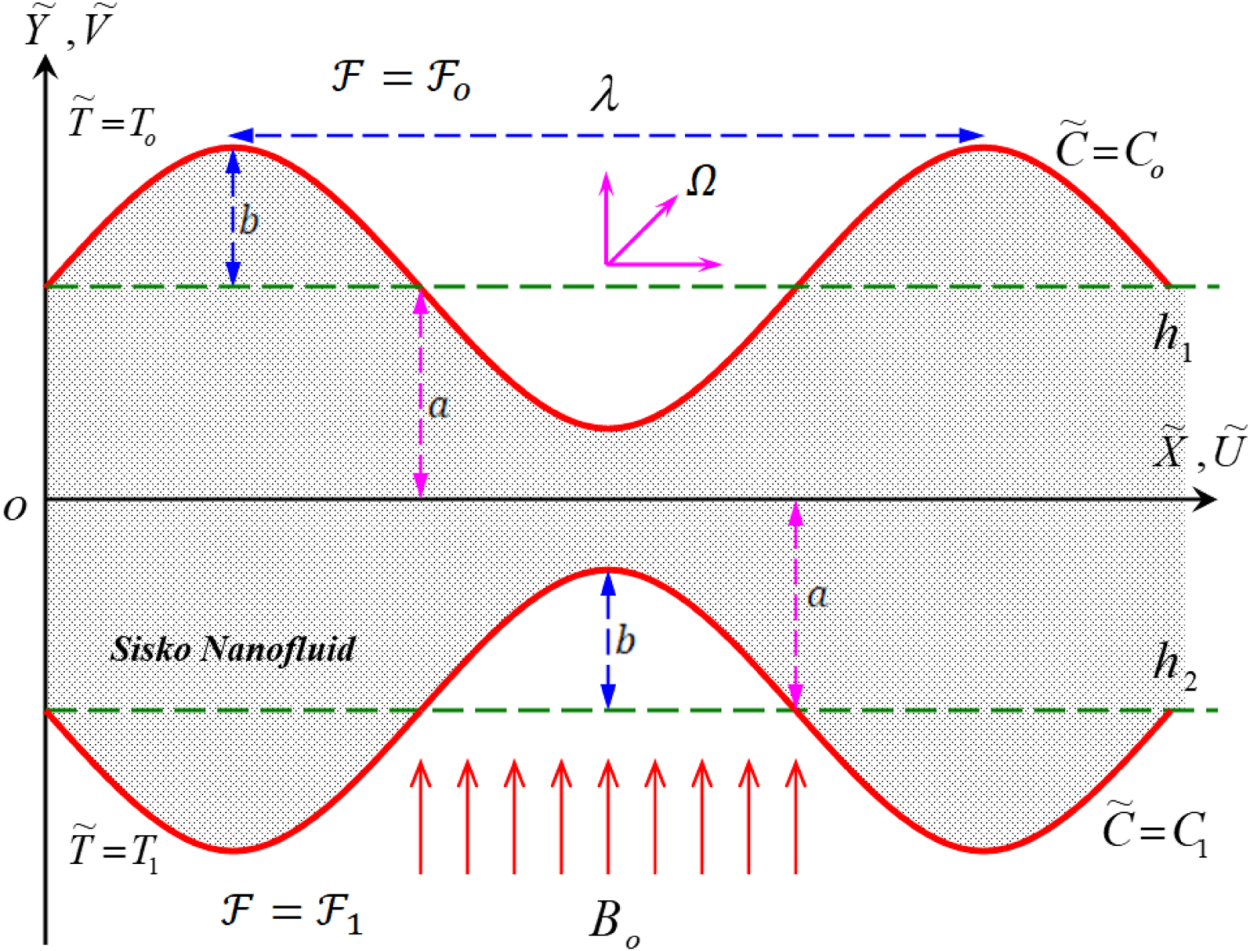
The geometry of the problem.

To begin with, the incompressible non-Newtonian Sisko nanofluid flow is a steady two-dimensional flow within a symmetric horizontal elastic channel. So that this fluid moves inside that channel a peristaltic movement through a porous medium under the influence of the magnetic field ${B}_{o}$ which in turn acts as an external force whose direction is perpendicular to the channel direction (in $\widetilde{Y}$ axis direction) On the other hand, the peristaltic flow is affected by the transmission of sine waves in the channel walls which move and advance at a constant speed $c$ in the direction of the $\widetilde{X}$ axis. Considering the effects which are Ohmic heating, non-linear thermal radiation, heat generation, and absorption, the Brownian motion, thermophoresis, and finally the boundary convective conditions in presence of viscous dissipation. Figure 1 represents the geometry of the channel that used in the current study and the upper and lower channel boundaries for that identical channel are:

1 $$\tilde{h}_{1} \left( {\tilde{X},\tilde{t}} \right) = b + a\sin \frac{2\pi }{\lambda }\left( {\tilde{X} - c\tilde{t}} \right)$$

2 $$\tilde{h}_{2} \left( {\tilde{X},\tilde{t}} \right) = - b - a\sin \frac{2\pi }{\lambda }\left( {\tilde{X} - c\tilde{t}} \right).$$

The velocity field in the two-dimensional flow can be written as follows^38^:

3 $$\vec{q} = \left( {\tilde{U}\left( {\tilde{X},\tilde{Y},\tilde{t}} \right),\left( {\tilde{V}\left( {\tilde{X},\tilde{Y},\tilde{t}} \right),0} \right)} \right).$$

The governing equations of the non-Newtonian nanofluid motion can be written in the mathematical formula as follows:

### The continuity equation

The continuity equation for the conservation of mass becomes^34,38^:

4 $$\nabla \cdot \vec{q} = 0.$$

### The momentum equation

The Navier–Stokes equation for the balance of momentum is given by^38^:

5 $$\rho \frac{{\partial \vec{q}}}{{\partial \tilde{t}}} + \rho \left( {\begin{array}{*{20}c} {\vec{\Omega } \times \left( {\vec{\Omega } \times \vec{q}} \right)} \\ { + 2\vec{\Omega } \times \vec{q}} \\ \end{array} } \right) = div\left( {{\mathbf{\overline{\rm T}}}} \right) + \overrightarrow {{F_{e} }} - \frac{\mu }{{K_{P} }}\vec{q} + \left( {1 - C_{o} } \right)\rho g\left( {\begin{array}{*{20}c} {\beta_{T} \left( {\tilde{T} - T_{o} } \right)} \\ { + \beta_{{\mathcal{F}}} \left( {\widetilde{{\mathcal{F}}} - {\mathcal{F}}_{o} } \right)} \\ \end{array} } \right) - g\left( {\rho_{p} - \rho } \right)\left( {\tilde{C} - C_{o} } \right),$$

where $$\overrightarrow {{F_{e} }} = \vec{J} \times \vec{B}$$, $$\vec{J} = \sigma \left( {\vec{E} + \vec{q} \times \vec{B}} \right)$$ and $$\vec{q} = \left( {u, v, 0} \right)$$.

### The energy equation

The conservation of energy for heat transfer is given by the constitutive equation as^7,38,49^:

6 $$\left( {\rho c_{P} } \right)_{f} \left( {\begin{array}{*{20}c} {\frac{{\partial \tilde{T}}}{{\partial \tilde{t}}}} \\ { + \vec{q} \cdot \nabla \tilde{T}} \\ \end{array} } \right) = \nabla \cdot \left( {\begin{array}{*{20}c} {k\nabla \tilde{T} + {q}_{r} } \\ { + D_{{T{\mathcal{F}}}} \nabla \widetilde{{\mathcal{F}}}} \\ \end{array} } \right) + \left( {\rho c_{P} } \right)_{p} \left( {\begin{array}{*{20}c} {D_{B} \nabla \tilde{C} \cdot \nabla \tilde{T}} \\ { + D_{T} \frac{{\nabla \tilde{T} \cdot \nabla \tilde{T}}}{{T_{o} }}} \\ \end{array} } \right) + Q_{o} \left( {\tilde{T} - T_{o} } \right) + \mu \nabla q^{2} + J_{he} ,$$

where $$J_{he} = \vec{F}_{e} \cdot \vec{q}$$.

### The solutal concentration equation

The conservation of solutal concentration is given by the constitutive equation as^7^

7 $$\frac{{\partial \widetilde{{\mathcal{F}}}}}{\partial t} + \vec{q} \cdot \nabla \widetilde{{\mathcal{F}}} = \nabla \cdot \left( {D_{S} \nabla \widetilde{{\mathcal{F}}} + D_{{{\mathcal{F}}T}} \nabla \tilde{T}} \right).$$

### The nanoparticles volume fraction equation

The nanoparticles volume fraction equation can be written as the mathematical formula as follows^7,38^:

8 $$\frac{{\partial \tilde{C}}}{\partial t} + \vec{q} \cdot \nabla \tilde{C} = \nabla \cdot \left( {D_{B} \nabla \tilde{C} + D_{T} \frac{{\nabla \tilde{T}}}{{T_{o} }}} \right).$$

The Cauchy stress tensor $\overline{\mathbf{\rm T} }$ for the non-Newtonian Sisko fluid can be written as follows^7,12^:

9 $${\mathbf{\overline{\rm T}}} = - P\overline{I} + \left( {\alpha^{*} + \beta^{**} \left( {\sqrt \Pi } \right)^{n - 1} } \right)\overline{A}_{1} , \Pi = \frac{1}{2}tr\left( {\overline{A}_{1} } \right)^{2} .$$

The Rivlin–Ericksen tensor $\overline{A}_{i} { }\left( {i = 1{ }to{ }4} \right)$ can be defined as follows^12^:

10 $${ }\overline{A}_{1} = \left( {\nabla \vec{q}} \right) + \left( {\nabla \vec{q}} \right)^{{\tilde{\tau }}} ,$$

11 $$\overline{A}_{i} = \frac{d}{dt}\overline{A}_{i - 1} + \overline{A}_{i - 1} \left( {\nabla \vec{q}} \right) + \left( {\nabla \vec{q}} \right)^{{\tilde{\tau }}} \overline{A}_{i - 1} , i = 2,3,4.$$

Power index $$n$$ provides information about shear thinning $$(n<1)$$ and shear thickening $$(n>1)$$ nature of fluid. Further $${\alpha }^{*}=0$$, the Sisko model is converted into generalized power law model. Further note that for ($$n=1$$, $${\alpha }^{*}=0$$, $${\beta }^{**}=\mu$$) or ($${\alpha }^{*}=\mu$$, $${\beta }^{**}=0$$), the viscous fluid model is recovered.

Considering the velocity field for the proposed problem as $\overrightarrow{q}=\left(\widetilde{U}\left(\widetilde{X},\widetilde{Y},\widetilde{t}\right),\widetilde{V}\left(\widetilde{X},\widetilde{Y},\widetilde{t}\right),0\right)$, so Eqs. (4)–(8) are defined in the laboratory frame $(\widetilde{X},\widetilde{Y})$ as follows^7^:

12 $$\frac{{\partial \tilde{U}}}{{\partial \tilde{X}}} + \frac{{\partial \tilde{V}}}{{\partial \tilde{Y}}} = 0,$$

13 $$ \begin{aligned}&\rho \left(\frac{\partial \widetilde{U}}{\partial \widetilde{t}}+\widetilde{U}\frac{\partial \widetilde{U}}{\partial \widetilde{X}}+\widetilde{V}\frac{\partial \widetilde{U}}{\partial \widetilde{Y}}\right)=-\frac{\partial \widetilde P}{\partial \widetilde{X}}+\rho\Omega \left(\Omega \widetilde{U}+2\frac{\partial \widetilde{V}}{\partial \widetilde{t}}\right)\\ &\quad+\frac{\partial {\overline{S} }_{XX}}{\partial \widetilde{X}}+\frac{\partial {\overline{S} }_{XY}}{\partial \widetilde{Y}}+\rho g\left(1-{C}_{o}\right)\left(\begin{array}{c}{\beta }_{T}\left(\widetilde{T}-{T}_{o}\right)\\ +{\beta }_{c}\left(\widetilde{\mathcal{F}}-{\mathcal{F}}_{o}\right)\end{array}\right)\\ &\quad-g\left({\rho }_{p}-\rho \right)\left(\widetilde{C}-{C}_{o}\right)-\frac{\mu }{{K}_{p}}\widetilde{U}-\sigma {B}_{o}^{2}\widetilde{U},\end{aligned} $$

14 $$\rho \left( {\frac{{\partial \tilde{V}}}{{\partial \tilde{t}}} + \tilde{U}\frac{{\partial \tilde{V}}}{{\partial \tilde{X}}} + \tilde{V}\frac{{\partial \tilde{V}}}{{\partial \tilde{X}}}} \right) = - \frac{\partial \widetilde P}{{\partial \tilde{Y}}} + \rho {\Omega }\left( {{\Omega }\tilde{V} - 2\frac{{\partial \tilde{U}}}{{\partial \tilde{t}}}} \right) + \frac{{\partial \overline{S}_{YX} }}{{\partial \tilde{X}}} + \frac{{\partial \overline{S}_{YY} }}{{\partial \tilde{Y}}}-\frac{\mu }{{K}_{p}}\widetilde{V},$$

15 $$ \begin{aligned} & {\left(\rho {c}_{P}\right)}_{f}\left(\frac{\partial \widetilde{T}}{\partial \widetilde{t}}+\widetilde{U}\frac{\partial \widetilde{T}}{\partial \widetilde{X}}+\widetilde{V}\frac{\partial \widetilde{T}}{\partial \widetilde{Y}}\right)=k\left(\begin{array}{c}\frac{{\partial }^{2}\widetilde{T}}{\partial {\widetilde{X}}^{2}}\\ +\frac{{\partial }^{2}\widetilde{T}}{\partial {\widetilde{Y}}^{2}}\end{array}\right)\\&\quad+{\left(\rho {c}_{P}\right)}_{p}\left(\begin{array}{c}{D}_{B}\left(\frac{\partial \widetilde{C}}{\partial \widetilde{X}}\frac{\partial \widetilde{T}}{\partial \widetilde{X}}+\frac{\partial \widetilde{C}}{\partial \widetilde{Y}}\frac{\partial \widetilde{T}}{\partial \widetilde{Y}}\right)\\ +\left(\frac{{D}_{T}}{{T}_{\infty }}\right)\left({\left(\frac{\partial \widetilde{T}}{\partial \widetilde{X}}\right)}^{2}+{\left(\frac{\partial \widetilde{T}}{\partial \widetilde{Y}}\right)}^{2}\right)\end{array}\right)+{D}_{T\mathcal{F}}\left(\frac{{\partial }^{2}\widetilde{\mathcal{F}}}{\partial {\widetilde{X}}^{2}}+\frac{{\partial }^{2}\widetilde{\mathcal{F}}}{\partial {\widetilde{Y}}^{2}}\right)+{Q}_{o}\left(\widetilde{T}-{T}_{o}\right)\\&\quad +\sigma {B}_{o}^{2}{\widetilde{U}}^{2}+\mu {\left(\frac{\partial \widetilde{u}}{\partial \widetilde{Y}}\right)}^{2}-\frac{\partial {q}_{r}}{\partial \widetilde{Y}},\end{aligned} $$

16 $$\frac{{\partial \widetilde{{\mathcal{F}}}}}{{\partial \tilde{t}}} + \tilde{U}\frac{{\partial \widetilde{{\mathcal{F}}}}}{{\partial \tilde{X}}} + \tilde{V}\frac{{\partial \widetilde{{\mathcal{F}}}}}{{\partial \tilde{Y}}} = D_{S} \left( {\frac{{\partial^{2} \widetilde{{\mathcal{F}}}}}{{\partial \tilde{X}^{2} }} + \frac{{\partial^{2} \widetilde{{\mathcal{F}}}}}{{\partial \tilde{Y}^{2} }}} \right) + D_{{{\mathcal{F}}T}} \left( {\frac{{\partial^{2} \tilde{T}}}{{\partial \tilde{X}^{2} }} + \frac{{\partial^{2} \tilde{T}}}{{\partial \tilde{Y}^{2} }}} \right),$$

17 $$\frac{{\partial \tilde{C}}}{{\partial \tilde{t}}} + \tilde{U}\frac{{\partial \tilde{C}}}{{\partial \tilde{X}}} + \tilde{V}\frac{{\partial \tilde{C}}}{{\partial \tilde{Y}}} = D_{B} \left( {\frac{{\partial^{2} \tilde{C}}}{{\partial \tilde{X}^{2} }} + \frac{{\partial^{2} \tilde{C}}}{{\partial \tilde{Y}^{2} }}} \right) + \left( {\frac{{D_{T} }}{{T_{o} }}} \right)\left( {\frac{{\partial^{2} \tilde{T}}}{{\partial \tilde{X}^{2} }} + \frac{{\partial^{2} \tilde{T}}}{{\partial \tilde{Y}^{2} }}} \right),$$

where the extra stress tensors $\overline S_{XX} ,\overline S_{XY}$ and $\overline S_{YY}$ can be written as follows^7^

18 $$\overline S_{XX} = 2\frac{{\partial \tilde{U}}}{{\partial \tilde{X}}}\left( {\hat{\mu } + \eta \left( {2\left( {\frac{{\partial \tilde{U}}}{{\partial \tilde{X}}}} \right)^{2} + \left( {\frac{{\partial \tilde{U}}}{{\partial \tilde{Y}}} + \frac{{\partial \tilde{V}}}{{\partial \tilde{X}}}} \right)^{2} + 2\left( {\frac{{\partial \tilde{V}}}{{\partial \tilde{Y}}}} \right)^{2} } \right)^{{\frac{n - 1}{2}}} } \right),$$

19 $$\overline S_{XY} = \left( {\frac{{\partial \tilde{U}}}{{\partial \tilde{Y}}} + \frac{{\partial \tilde{V}}}{{\partial \tilde{X}}}} \right)\left( {\hat{\mu } + \eta \left( {2\left( {\frac{{\partial \tilde{U}}}{{\partial \tilde{X}}}} \right)^{2} + \left( {\frac{{\partial \tilde{U}}}{{\partial \tilde{Y}}} + \frac{{\partial \tilde{V}}}{{\partial \tilde{X}}}} \right)^{2} + 2\left( {\frac{{\partial \tilde{V}}}{{\partial \tilde{Y}}}} \right)^{2} } \right)^{{\frac{n - 1}{2}}} } \right),$$

20 $$\overline S_{YY} = 2\frac{{\partial \tilde{V}}}{{\partial \tilde{Y}}}\left( {\hat{\mu } + \eta \left( {2\left( {\frac{{\partial \tilde{U}}}{{\partial \tilde{X}}}} \right)^{2} + \left( {\frac{{\partial \tilde{U}}}{{\partial \tilde{Y}}} + \frac{{\partial \tilde{V}}}{{\partial \tilde{X}}}} \right)^{2} + 2\left( {\frac{{\partial \tilde{V}}}{{\partial \tilde{Y}}}} \right)^{2} } \right)^{{\frac{n - 1}{2}}} } \right).$$

In fixed frames $\left( {\tilde{X},\tilde{Y}} \right)$, the flow process is unsteady, but in wave frames $\left( {\tilde{x}, \tilde{y}} \right)$ the motion process is steady. Thus, the transformative relationship that links the two frames together can be listed as follows:

21 $$\tilde{x} = \tilde{X} - c\tilde{t}, \tilde{y} = \tilde{Y}, \tilde{u}\left( {\tilde{x},\tilde{y}} \right) = \tilde{U} - c, \tilde{v}\left( {\tilde{x},\tilde{y}} \right) = \tilde{V}.$$

The basic governing partial differential equations of the MHD non-Newtonian Sisko nanofluid (12) – (17) using the Eqs. (9) and (10) and the transformation Eqs. (21) can be written as mathematically follows;

22 $$\frac{{\partial \tilde{u}}}{{\partial \tilde{x}}} + \frac{{\partial \tilde{v}}}{{\partial \tilde{y}}} = 0,$$

23 $$\rho \left(\widetilde{u}\frac{\partial \widetilde{u}}{\partial \widetilde{x}}+\widetilde{v}\frac{\partial \widetilde{u}}{\partial \widetilde{y}}\right)=-\frac{\partial \widetilde {P}}{\partial \widetilde{x}}+\rho {\Omega }^{2}\widetilde{u}+\frac{\partial {\overline{S} }_{xx}}{\partial\widetilde x}+\frac{\partial {\overline{S} }_{xy}}{\partial\widetilde y}+\rho g\left(1-{C}_{o}\right)\left(\begin{array}{c}{\beta }_{T}\left(\widetilde{T}-{T}_{o}\right)\\ +{\beta }_{c}\left(\widetilde{\mathcal{F}}-{\mathcal{F}}_{o}\right)\end{array}\right)-g\left({\rho }_{p}-\rho \right)\left(\widetilde{C}-{C}_{o}\right)-\frac{\mu }{{K}_{p}}\widetilde{u}-\sigma {B}_{o}^{2}\widetilde{u},$$

24 $$\rho \left( {\tilde{u}\frac{{\partial \tilde{v}}}{{\partial \tilde{x}}} + \tilde{v}\frac{{\partial \tilde{v}}}{{\partial \tilde{y}}}} \right) = - \frac{\partial \tilde{P}}{{\partial \tilde{y}}} + \rho {\Omega }^{2} \tilde{v} + \frac{{\partial \overline{S}_{xy} }}{{\partial \tilde{x}}} + \frac{{\partial \overline{S}_{yy} }}{{\partial \tilde{y}}}-\frac{\mu }{{K}_{p}}\widetilde{v},$$

25 $$ \begin{aligned} & {\left(\rho {c}_{P}\right)}_{f}\left(\widetilde{u}\frac{\partial \widetilde{T}}{\partial \widetilde{x}}+\widetilde{v}\frac{\partial \widetilde{T}}{\partial \widetilde{y}}\right)=k\left(\frac{{\partial }^{2}\widetilde{T}}{\partial {\widetilde{x}}^{2}}+\frac{{\partial }^{2}\widetilde{T}}{\partial {\widetilde{y}}^{2}}\right)+{\left(\rho {c}_{P}\right)}_{p}\left({D}_{B}\left(\frac{\partial \widetilde{C}}{\partial \widetilde{x}}\frac{\partial \widetilde{T}}{\partial \widetilde{x}}+\frac{\partial \widetilde{C}}{\partial \widetilde{y}}\frac{\partial \widetilde{T}}{\partial \widetilde{y}}\right)+\left(\frac{{D}_{T}}{{T}_{\infty }}\right)\left({\left(\frac{\partial \widetilde{T}}{\partial \widetilde{x}}\right)}^{2}+{\left(\frac{\partial \widetilde{T}}{\partial \widetilde{y}}\right)}^{2}\right)\right)\\ &\quad+{D}_{T\mathcal{F}}\left(\frac{{\partial }^{2}\widetilde{\mathcal{F}}}{\partial {\widetilde{x}}^{2}}+\frac{{\partial }^{2}\widetilde{\mathcal{F}}}{\partial {\widetilde{y}}^{2}}\right)+{Q}_{o}\left(\widetilde{T}-{T}_{o}\right)+\sigma {B}_{o}^{2}{\widetilde{u}}^{2}+\mu {\left(\frac{\partial \widetilde{u}}{\partial \widetilde{y}}\right)}^{2}-\frac{\partial {q}_{r}}{\partial \widetilde{y}},\end{aligned} $$

26 $$\tilde{u}\frac{{\partial \widetilde{{\mathcal{F}}}}}{{\partial \tilde{x}}} + \tilde{v}\frac{{\partial \widetilde{{\mathcal{F}}}}}{{\partial \tilde{y}}} = D_{S} \left( {\frac{{\partial^{2} \widetilde{{\mathcal{F}}}}}{{\partial \tilde{x}^{2} }} + \frac{{\partial^{2} \widetilde{{\mathcal{F}}}}}{{\partial \tilde{y}^{2} }}} \right) + D_{{{\mathcal{F}}T}} \left( {\frac{{\partial^{2} \tilde{T}}}{{\partial \tilde{x}^{2} }} + \frac{{\partial^{2} \tilde{T}}}{{\partial \tilde{y}^{2} }}} \right),$$

27 $$\tilde{u}\frac{{\partial \tilde{C}}}{{\partial \tilde{x}}} + \tilde{v}\frac{{\partial \tilde{C}}}{{\partial \tilde{y}}} = D_{B} \left( {\frac{{\partial^{2} \tilde{C}}}{{\partial \tilde{x}^{2} }} + \frac{{\partial^{2} \tilde{C}}}{{\partial \tilde{y}^{2} }}} \right) + \left( {\frac{{D_{T} }}{{T_{o} }}} \right)\left( {\frac{{\partial^{2} \tilde{T}}}{{\partial \tilde{x}^{2} }} + \frac{{\partial^{2} \tilde{T}}}{{\partial \tilde{y}^{2} }}} \right).$$

The appropriate boundary conditions may be written as:

28 $$\tilde{u} = 0,k\frac{{\partial \tilde{T}}}{\partial y} = - h_{2}^{*} \left( {T_{o} - \tilde{T}} \right),D_{m} \frac{{\partial \widetilde{{\mathcal{F}}}}}{\partial y} = - L_{2} \left( {{\mathcal{F}}_{o} - \widetilde{{\mathcal{F}}}} \right), C = C_{o} \;at \;y = - b - a\sin \frac{2\pi }{\lambda }\left( {\tilde{X} - c\tilde{t}} \right),$$

29 $$\tilde{u} = 0,k\frac{{\partial \tilde{T}}}{\partial y} = - h_{1}^{*} \left( {\tilde{T} - T_{1} } \right),D_{m} \frac{{\partial \widetilde{{\mathcal{F}}}}}{\partial y} = - L_{1} \left( {\widetilde{{\mathcal{F}}} - {\mathcal{F}}_{1} } \right), C = C_{1} \;at y = b + a\sin \frac{2\pi }{\lambda }\left( {\tilde{X} - c\tilde{t}} \right).$$

Using non-dimensional quantities as follows:

$$\tilde{x} = x\lambda ,\tilde{y} = d_{1} y,\tilde{t} = \lambda t/c,a = \delta \lambda ,\tilde{u} = cu,\tilde{v} = cv,\overline{S}_{ij} = \mu cS_{ij}/a,\tilde{P} = \mu\lambda cP/a^2,$$

$$k = \alpha \left( {\rho c_{p} } \right)_{f} ,Pr = \mu c_{P} /k,Re = ac\rho /\mu ,M = \sigma B_{o}^{2} a^{2} /\mu ,$$

$$\theta_{w} = T_{o} /\left( {T_{1} - T_{o} } \right),\,\beta = Q_{o} d_{1}^{2} /\mu c_{P} ,Da = a^{2} /K_{p} ,Bn = Pr \cdot Ec,$$

$$Bi_{1} = h_{1}^{*} a/K_{c} ,Bi_{2} = h_{2}^{*} a/K_{c} ,Mi_{1} = L_{1} a/D,Mi_{2} = L_{2} a/D,$$

$$Ec = c^{2} /c_{P} \left( {T_{1} - T_{o} } \right),Nb = \left( {\rho c_{P} } \right)_{p} D_{B} \left( {C_{1} - C_{o} } \right)/k,$$

$$\tilde{T} = T_{o} + \left( {T_{1} - T_{o} } \right)\theta ,Nt = \left( {\rho c_{P} } \right)_{p} D_{T} \left( {T_{1} - T_{o} } \right)/T_{o} k,$$

$$\tilde{C} = C_{o} + \left( {C_{1} - C_{o} } \right)\Phi , \, R = 16\sigma^{*} \left( {T_{1} - T_{o} } \right)^{3} /3kk^{*} ,$$

$$\widetilde{{\mathcal{F}}} = {\mathcal{F}}_{o} + \left( {{\mathcal{F}}_{1} - {\mathcal{F}}_{o} } \right)\gamma ,Gr_{p} = \left( {\rho_{p} - \rho } \right)a^{2} g\beta_{c} \left( {C_{1} - C_{o} } \right)/c\mu ,$$

$$Gr_{t} = \left( {1 - C_{o} } \right)\rho a^{2} g\beta_{T} \left( {T_{1} - T_{o} } \right)/c\mu ,Gr_{c} = \left( {1 - C_{o} } \right)\rho a^{2} g\beta_{T} \left( {{\mathcal{F}}_{1} - {\mathcal{F}}_{o} } \right) /c\mu ,$$

$$N_{{{\mathcal{F}}T}} = D_{{{\mathcal{F}}T}} \left( {T_{1} - T_{o} } \right)/D_{S} \left( {{\mathcal{F}}_{1} - {\mathcal{F}}_{o} } \right),\;N_{{T{\mathcal{F}}}} = D_{{T{\mathcal{F}}}} \left( {{\mathcal{F}}_{1} - {\mathcal{F}}_{o} } \right)/k\left( {T_{1} - T_{o} } \right),$$

where $${q}_{r}=-4{\sigma }^{*}\left(\frac{\partial {\widetilde{T}}^{4}}{\partial \widetilde{y}}\right)/3{k}^{*}$$.

The velocity components $u$ and $v$ are defined using the stream function $\psi$ as follows:

30 $$u = \frac{\partial \psi }{{\partial y}}, v = - \delta \frac{\partial \psi }{{\partial x}}.$$

The continuity Eq. (22) has been satisfied and using the non-dimensional quantities and substituting Eq. (30) into the Eqs. (23)–(27) these equations take the new mathematical form as follows:

31 $$Re\delta \left(\frac{\partial \psi }{\partial y}\frac{{\partial }^{2}\psi }{\partial x\partial y}-\frac{\partial \psi }{\partial x}\frac{{\partial }^{2}\psi }{\partial {y}^{2}}\right)=-\frac{\partial P}{\partial x}+\frac{{\Omega }^{2}{a}^{2}\rho }{\mu }\frac{\partial \psi }{\partial y}+\delta \frac{\partial {S}_{xx}}{\partial x}+\frac{\partial {S}_{xy}}{\partial y}+{Gr}_{t}\theta +{Gr}_{c}\gamma -{Gr}_{p}\Phi -Da\frac{\partial \psi }{\partial y}-M\frac{\partial \psi }{\partial y},$$

32 $$Re\delta^{3} \left( {\frac{\partial \psi }{{\partial y}}\frac{{\partial^{2} \psi }}{\partial x\partial y} - \frac{\partial \psi }{{\partial x}}\frac{{\partial^{2} \psi }}{{\partial y^{2} }}} \right) = - \frac{\partial P}{{\partial y}} + \frac{{\Omega^{2}\delta^{2} a^{2} \rho }}{\mu }\frac{\partial \psi }{{\partial x}} + \delta \left( {\delta \frac{{\partial S_{xy} }}{\partial x} + \frac{{\partial S_{yy} }}{\partial y}} \right)+\frac{a^{2}\delta^{2} }{{K}_{p}}\frac{\partial \psi }{{\partial x}},$$

33 $$ \begin{aligned} & RePr\delta \left(\frac{\partial \psi }{\partial y}\frac{\partial \theta }{\partial x}-\frac{\partial \psi }{\partial x}\frac{\partial \theta }{\partial y}\right)=\left({\delta }^{2}\frac{{\partial }^{2}\theta }{\partial {x}^{2}}+\frac{{\partial }^{2}\theta }{\partial {y}^{2}}\right)+NbPr\left({\delta }^{2}\frac{\partial \theta }{\partial x}\frac{\partial\Phi }{\partial x}+\frac{\partial \theta }{\partial y}\frac{\partial\Phi }{\partial y}\right)+BnM{\left(\frac{\partial \psi }{\partial y}\right)}^{2}+Pr\beta \theta \\&\quad+NtPr\left({\delta }^{2}{\left(\frac{\partial \theta }{\partial x}\right)}^{2}+{\left(\frac{\partial \theta }{\partial y}\right)}^{2}\right)+{N}_{T\mathcal{F}}Pr\left({\delta }^{2}\frac{{\partial }^{2}\gamma }{\partial {x}^{2}}+\frac{{\partial }^{2}\gamma }{\partial {y}^{2}}\right)+EcPr{\left(\frac{{\partial }^{2}\psi }{\partial {y}^{2}}\right)}^{2}+PrR{\left({\left({\theta }_{w}+\theta \right)}^{3}\left(\frac{\partial \theta }{\partial y}\right)\right)}^{^{\prime}},\end{aligned} $$

34 $$Re\delta \left( {\frac{\partial \psi }{{\partial y}}\frac{{\partial {\upgamma }}}{\partial x} - \frac{\partial \psi }{{\partial x}}\frac{{\partial {\upgamma }}}{\partial y}} \right) = \left( {\delta^{2} \frac{{\partial^{2} {\upgamma }}}{{\partial x^{2} }} + \frac{{\partial^{2} {\upgamma }}}{{\partial y^{2} }}} \right) + N_{{{\mathcal{F}}T}} \left( {\delta^{2} \frac{{\partial^{2} \theta }}{{\partial x^{2} }} + \frac{{\partial^{2} \theta }}{{\partial y^{2} }}} \right),$$

35 $$Re\delta \left( {\frac{\partial \psi }{{\partial y}}\frac{{\partial {\Phi }}}{\partial x} - \frac{\partial \psi }{{\partial x}}\frac{{\partial {\Phi }}}{\partial y}} \right) = \left( {\delta^{2} \frac{{\partial^{2} {\Phi }}}{{\partial x^{2} }} + \frac{{\partial^{2} {\Phi }}}{{\partial y^{2} }}} \right) + \frac{Nt}{{Nb}}\left( {\delta^{2} \frac{{\partial^{2} \theta }}{{\partial x^{2} }} + \frac{{\partial^{2} \theta }}{{\partial y^{2} }}} \right),$$

where:

36 $$S_{xx} = 2\delta \left( {1 + \eta \left( {2\delta^{2} \left( {\frac{{\partial^{2} \psi }}{\partial x\partial y}} \right)^{2} + \left( {\frac{{\partial^{2} \psi }}{{\partial y^{2} }} - \delta^{2} \frac{{\partial^{2} \psi }}{{\partial x^{2} }}} \right)^{2} + 2\delta^{2} \left( {\frac{{\partial^{2} \psi }}{\partial x\partial y}} \right)^{2} } \right)^{{\frac{n - 1}{2}}} } \right)\frac{{\partial^{2} \psi }}{\partial x\partial y},$$

37 $$S_{xy} = \left( {1 + \eta \left( {2\delta^{2} \left( {\frac{{\partial^{2} \psi }}{\partial x\partial y}} \right)^{2} + \left( {\frac{{\partial^{2} \psi }}{{\partial y^{2} }} - \delta^{2} \frac{{\partial^{2} \psi }}{{\partial x^{2} }}} \right)^{2} + 2\delta^{2} \left( {\frac{{\partial^{2} \psi }}{\partial x\partial y}} \right)^{2} } \right)^{{\frac{n - 1}{2}}} } \right)\left( {\frac{{\partial^{2} \psi }}{{\partial y^{2} }} - \delta^{2} \frac{{\partial^{2} \psi }}{{\partial x^{2} }}} \right)^{2} ,$$

38 $$S_{xx} = - 2\delta \left( {1 + \eta \left( {2\delta^{2} \left( {\frac{{\partial^{2} \psi }}{\partial x\partial y}} \right)^{2} + \left( {\frac{{\partial^{2} \psi }}{{\partial y^{2} }} - \delta^{2} \frac{{\partial^{2} \psi }}{{\partial x^{2} }}} \right)^{2} + 2\delta^{2} \left( {\frac{{\partial^{2} \psi }}{\partial x\partial y}} \right)^{2} } \right)^{{\frac{n - 1}{2}}} } \right)\frac{{\partial^{2} \psi }}{\partial x\partial y}.$$

Using the long-wavelength approximation and neglecting the wave number along with the low Reynolds number accordingly, the final forms of the Eqs. (31)–(35) become as follows;

39 $$\frac{\partial P}{{\partial x}} = \frac{{\Omega^{2} a^{2} \rho }}{\mu }\frac{\partial \psi }{{\partial y}} + \frac{{\partial S_{xy} }}{\partial y} + Gr_{t} \theta + Gr_{c} \gamma - Gr_{p} {\Phi } - Da\frac{\partial \psi }{{\partial y}} - M\frac{\partial \psi }{{\partial y}},$$

40 $$\frac{\partial P}{{\partial y}} = 0,$$

41 $$ \begin{aligned}\frac{{\partial^{2} \theta }}{{\partial y^{2} }} & = - \left( {Pr Nb\left( {\frac{\partial \theta }{{\partial y}}\frac{{\partial {\Phi }}}{\partial y}} \right) + Bn M\left( {\frac{\partial \psi }{{\partial y}}} \right)^{2} + \beta Pr \theta + Nt Pr\left( {\frac{\partial \theta }{{\partial y}}} \right)^{2} }\right.\\&\quad\left.{+ Pr N_{{T{\mathcal{F}}}} \left( {\frac{{\partial^{2} \gamma }}{{\partial y^{2} }}} \right) + Ec Pr\left( {\frac{{\partial^{2} \psi }}{{\partial y^{2} }}} \right)^{2} + R Pr\left( {\left( {\theta_{w} + \theta } \right)^{3} \left( {\frac{\partial \theta }{{\partial y}}} \right)} \right)^{^{\prime}} } \right),\end{aligned} $$

42 $$\frac{{\partial^{2} \gamma }}{{\partial y^{2} }} = - N_{{{\mathcal{F}}T}} \left( {\frac{{\partial^{2} \theta }}{{\partial y^{2} }}} \right),$$

43 $$\frac{{\partial^{2} {\Phi }}}{{\partial y^{2} }} = - \frac{Nt}{{Nb}}\left( {\frac{{\partial^{2} \theta }}{{\partial y^{2} }}} \right),$$

where $$S_{xx} = 0, S_{yy} = 0$$ and

44 $$S_{xy} = \left( {1 + \eta \left( {\left( {\frac{{\partial^{2} \psi }}{{\partial y^{2} }}} \right)^{2} } \right)^{{\frac{n - 1}{2}}} } \right)\frac{{\partial^{2} \psi }}{{\partial y^{2} }} = \frac{{\partial^{2} \psi }}{{\partial y^{2} }} + \eta \left( {\frac{{\partial^{2} \psi }}{{\partial y^{2} }}} \right)^{n} .$$

Substituting Eq. (44) into Eq. (39) results in:

45 $$\frac{\partial P}{{\partial x}} = \frac{{\Omega^{2} a^{2} \rho }}{\mu }\frac{\partial \psi }{{\partial y}} + \frac{\partial }{\partial y}\left( {\frac{{\partial^{2} \psi }}{{\partial y^{2} }} + \eta \left( {\frac{{\partial^{2} \psi }}{{\partial y^{2} }}} \right)^{n} } \right) + Gr_{t} \theta + Gr_{c} \gamma - Gr_{p} {\Phi } - Da\frac{\partial \psi }{{\partial y}} - M\frac{\partial \psi }{{\partial y}}.$$

By removing the pressure from Eqs. (40) and (45), it results:

46 $$\frac{{\partial^{4} \psi }}{{\partial y^{4} }}\left( {1 + n\eta \left( {\frac{{\partial^{2} \psi }}{{\partial y^{2} }}} \right)^{n - 1} } \right) - \left( {\begin{array}{*{20}c} {M\frac{{\partial^{2} \psi }}{{\partial y^{2} }} + Da\frac{{\partial^{2} \psi }}{{\partial y^{2} }} - Gr_{t} \frac{\partial \theta }{{\partial y}} - Gr_{c} \frac{\partial \gamma }{{\partial y}}} \\ { + Gr_{p} \frac{{\partial {\Phi }}}{\partial y} - \frac{{\rho {\Omega }^{2} a^{2} }}{\mu }\frac{{\partial^{2} \psi }}{{\partial y^{2} }} - n\left( {n - 1} \right)\eta \left( {\frac{{\partial^{3} \psi }}{{\partial y^{3} }}} \right)^{2} \left( {\frac{{\partial^{2} \psi }}{{\partial y^{2} }}} \right)^{n - 2} } \\ \end{array} } \right) = 0.$$

The boundary conditions (28) and (29) of the current problem in the wave frame are defined as follows

47 $$\psi = - \frac{F}{2}, \frac{\partial \psi }{{\partial y}} = 0, \frac{\partial \theta }{{\partial y}} = Bi_{2} \theta , \frac{\partial \gamma }{{\partial y}} = Mi_{2} \gamma , {\Phi } = 0, \;at \;y = h_{2} = - 1 - \varepsilon \sin 2\pi x,$$

48 $$\psi = \frac{F}{2}, \frac{\partial \psi }{{\partial y}} = 0, \frac{\partial \theta }{{\partial y}} = Bi_{1} \left( {1 - \theta } \right), \frac{\partial \gamma }{{\partial y}} = Mi_{1} \left( {1 - \gamma } \right), {\Phi } = 0, at y = h_{1} = 1 + \varepsilon \sin 2\pi x.$$

The dimensionless mean flow rate $Q$ in the laboratory frame is related to the dimensionless mean flow rate $F$ in the wave frame by $Q=1+F+a$ and $F=\underset{{h}_{2}}{\overset{{h}_{1}}{\int }}udy$ refer to the mean flow rate.

## Numerical solutions

The Runge–Kutta numerical method is one of the most important numerical analysis methods that are used in solving a system of ordinary differential equations, there are different formulas for the solution by the numerical method of Runge–Kutta method; for example, there is the Runge–Kutta method of the fourth order and also the Runge–Kutta method of the fifth order, and the most used method is the Runge–Kutta method of the fourth order because it gives accurate results and is easy to use, and its derivation depends on the Euler method. The numerical solutions of the current study are listed in several steps. It was previously mentioned that the system of differential Eqs. (13)–(17) that governs the peristaltic flow process within the symmetric horizontal channel are non-linear partial differential equations in fixed frames that have been converted to the system of non-linear partial differential Eqs. (23)–(26) with the boundary conditions (28) and (29) in the wave frames using transformations (21) as well as using the long-wavelength approximation with the small Reynolds number transforming the system of non-linear partial differential Eqs. (23)–(26) to the system of ordinary differential equations non-linearity (39)–(43) with new boundary terms (47) and (48) Finally, after deleting the pressure from Eqs. (39) and (40), we have the system of non-linear ordinary differential Eqs. (41)–(43) and (46), which has been solved by the best numerical methods, which is the fourth-order Runge Kutta method with the shooting technique by numerical code in the MATLAB program. Our equations are dealt with in MATLAB because each nth-order equation is mutated into *n* of the first-order equations, then using the bvp4c function (in general, bvp4c is a finite difference code that implements the three-stage lobatto IIIa formula. This is a collocation formula and the collocation polynomial provides a C1-continuous solution that is fourth-order accurate uniformly in $[a,b]$. Mesh selection and error control are based on the residual of the continuous solution) to solve these first-order equations when $\Delta y=0.01$ and the channel limits are $-1.5\le y\le 1.5$, it has been obtained by substituting in ${h}_{2}$ and ${h}_{1}$ in the boundary conditions (47) and (48) by $\varepsilon =0.5$ and $x=0.25$. The previous system of Eqs. (41)–(43) and (46) in its simplest form can be written as follows;

49 $$\frac{{\partial^{4} \psi }}{{\partial y^{4} }} = \left( {\begin{array}{*{20}c} {M\frac{{\partial^{2} \psi }}{{\partial y^{2} }} + Da\frac{{\partial^{2} \psi }}{{\partial y^{2} }} - Gr_{t} \frac{\partial \theta }{{\partial y}} - Gr_{c} \frac{\partial \gamma }{{\partial y}}} \\ { + Gr_{p} \frac{{\partial {\Phi }}}{\partial y} - \frac{{\rho {\Omega }^{2} a^{2} }}{\mu }\frac{{\partial^{2} \psi }}{{\partial y^{2} }} - n\left( {n - 1} \right)\eta \left( {\frac{{\partial^{3} \psi }}{{\partial y^{3} }}} \right)^{2} \left( {\frac{{\partial^{2} \psi }}{{\partial y^{2} }}} \right)^{n - 2} } \\ \end{array} } \right)/\left( {1 + n\eta \left( {\frac{{\partial^{2} \psi }}{{\partial y^{2} }}} \right)^{n - 1} } \right),$$

50 $$\frac{{\partial^{2} \theta }}{{\partial y^{2} }} = - \left( {Nb Pr\left( {\frac{\partial \theta }{{\partial y}}\frac{{\partial {\Phi }}}{\partial y}} \right) + Bn M\left( {\frac{\partial \psi }{{\partial y}}} \right)^{2} + \beta Pr \theta + Nt Pr\left( {\frac{\partial \theta }{{\partial y}}} \right)^{2} + Ec Pr\left( {\frac{{\partial^{2} \psi }}{{\partial y^{2} }}} \right) + 3R Pr\left( {\theta_{w} + \theta } \right)^{2} \left( {\frac{\partial \theta }{{\partial y}}} \right)^{2} } \right)/\left( {1 - Pr N_{{{\mathcal{F}}T}} N_{{T{\mathcal{F}}}} + R Pr\left( {\theta_{w} + \theta } \right)^{3} } \right),$$

51 $$\frac{{\partial^{2} \gamma }}{{\partial y^{2} }} = - N_{{{\mathcal{F}}T}} \left( {\frac{{\partial^{2} \theta }}{{\partial y^{2} }}} \right),$$

52 $$\frac{{\partial^{2} {\Phi }}}{{\partial y^{2} }} = - \frac{Nt}{{Nb}}\left( {\frac{{\partial^{2} \theta }}{{\partial y^{2} }}} \right).$$

The system of Eqs. (49)–(52) has been entered into the MATLAB code by degrading the differential order using new variables as follows:

$$\mathcal{A}(1)= \mathcal{Y}(2);$$

$$\mathcal{A}\left(2\right)= \mathcal{Y}(3);$$

$$\mathcal{A}(3)= \mathcal{Y}(4);$$

$$\mathcal{A}(4)= \left(\begin{array}{c}M Y\left(3\right)+Da Y\left(3\right)-{Gr}_{t} Y\left(6\right)-{Gr}_{c} Y\left(8\right)+{Gr}_{p} Y\left(10\right)\\ -\rho {\Omega }^{2} {a}^{2}\frac{\mathcal{Y}\left(3\right)}{\mu }-n\left(n-1\right)\eta {\left(\mathcal{Y}\left(4\right)\right)}^{2}{\left(\mathcal{Y}\left(3\right)\right)}^{\left(n-2\right)}\end{array}\right)/\left(1+n\eta {\left(\mathcal{Y}(3)\right)}^{(n-1)}\right);$$

$$\mathcal{A}(5)= \mathcal{Y}(6);$$

$$\mathcal{A}(6)= -\left(\begin{array}{c}NbPr Y\left(6\right) Y\left(10\right)+Nt{Pr\left( \mathcal{Y}\left(6\right)\right)}^{2}+\beta Pr Y\left(5\right)+BnM {\left(\mathcal{Y}\left(2\right)\right)}^{2}\\ +PrEc {\left(\mathcal{Y}\left(3\right)\right)}^{2}+3R{Pr\left({\theta }_{w}+\mathcal{Y}\left(5\right)\right)}^{2} {\left(\mathcal{Y}\left(6\right)\right)}^{2}\end{array}\right)/\left(\begin{array}{c}1-{N}_{\mathcal{F}T}{N}_{T\mathcal{F}}\\ +RPr{\left({\theta }_{w}+\mathcal{Y}\left(5\right)\right)}^{3}\end{array}\right);$$

$$\mathcal{A}(7)= \mathcal{Y}(8);$$

$$\mathcal{A}\left(8\right)= -{N}_{\mathcal{F}T} \mathcal{A}\left(6\right);$$

$$\mathcal{A}(9)= \mathcal{Y}(10);$$

$$\mathcal{A}\left(10\right)= -Nt \mathcal{A}(6)/Nb;$$

Also, the boundary conditions (47) and (48) were written inside the MATLAB code as follows;

$${\mathcal{Y}}_{a}\left(1\right)=-\frac{F}{2}, {\mathcal{Y}}_{a}\left(2\right)=0, {\mathcal{Y}}_{a}\left(6\right)={Bi}_{2} {\mathcal{Y}}_{a}\left(5\right), {\mathcal{Y}}_{a}\left(8\right)={Mi}_{2} {\mathcal{Y}}_{a}(7), {\mathcal{Y}}_{a}(9)=0, at y=-1.5$$

$${\mathcal{Y}}_{b}\left(1\right)=\frac{F}{2}, {\mathcal{Y}}_{b}\left(2\right)=0, {\mathcal{Y}}_{b}\left(6\right)={Bi}_{1}\left(1-{\mathcal{Y}}_{b}\left(5\right)\right), {\mathcal{Y}}_{b}\left(8\right)={Mi}_{1}\left(1-{\mathcal{Y}}_{b}(7)\right), {\mathcal{Y}}_{b}\left(9\right)=1, at y=1.5.$$

Taking into account the following;

$$\psi = \mathcal{Y}\left(1\right),\frac{\partial \psi }{\partial y}= \mathcal{Y}\left(2\right),\frac{{\partial }^{2}\psi }{\partial {y}^{2}}=\mathcal{Y}\left(3\right),\frac{{\partial }^{3}\psi }{\partial {y}^{3}}=\mathcal{Y}\left(4\right),\frac{{\partial }^{4}\psi }{\partial {y}^{4}}={\left(\mathcal{Y}\left(4\right)\right)}^{^{\prime}}=\mathcal{A}\left(4\right),\theta =\mathcal{Y}\left(5\right),\frac{\partial \theta }{\partial y}=\mathcal{Y}\left(6\right),$$

$$\frac{{\partial }^{2}\theta }{\partial {y}^{2}}={\left(\mathcal{Y}\left(6\right)\right)}^{^{\prime}}=\mathcal{A}\left(6\right),\gamma =\mathcal{Y}\left(7\right),\frac{\partial \gamma }{\partial y}=\mathcal{Y}\left(8\right),\frac{{\partial }^{2}\gamma }{\partial {y}^{2}}={\left(\mathcal{Y}\left(8\right)\right)}^{^{\prime}}=\mathcal{A}\left(8\right),\Phi =\mathcal{Y}\left(9\right),\frac{\partial\Phi }{\partial y}=\mathcal{Y}\left(10\right),$$

$$\frac{{\partial }^{2}\Phi }{\partial {y}^{2}}={\left(\mathcal{Y}\left(10\right)\right)}^{^{\prime}}=\mathcal{A}\left(10\right),{\mathcal{Y}}_{a}\left(1\right)={\psi }_{y=-1.5},{\mathcal{Y}}_{a}\left(2\right)={\left(\frac{\partial \psi }{\partial y}\right)}_{y=-1.5},{\mathcal{Y}}_{a}\left(5\right)={\theta }_{y=-1.5},$$

$${\mathcal{Y}}_{a}\left(6\right)={\left(\frac{\partial \theta }{\partial y}\right)}_{y=-1.5},{\mathcal{Y}}_{a}\left(7\right)={\gamma }_{y=-1.5},{\mathcal{Y}}_{a}\left(8\right)={\left(\frac{\partial \gamma }{\partial y}\right)}_{y=-1.5},{\mathcal{Y}}_{a}\left(9\right)={\Phi }_{y=-1.5},{\mathcal{Y}}_{b}\left(1\right)={\psi }_{y=1.5},$$

$${\mathcal{Y}}_{b}\left(2\right)={\left(\frac{\partial \psi }{\partial y}\right)}_{y=1.5},{\mathcal{Y}}_{b}\left(5\right)={\theta }_{y=1.5},{\mathcal{Y}}_{b}\left(6\right)={\left(\frac{\partial \theta }{\partial y}\right)}_{y=1.5},{\mathcal{Y}}_{b}\left(7\right)={\gamma }_{y=1.5},{\mathcal{Y}}_{b}\left(8\right)={\left(\frac{\partial \gamma }{\partial y}\right)}_{y=1.5}.$$

$${\mathcal{Y}}_{b}\left(9\right)={\Phi }_{y=1.5}.$$

## Results and discussion

In this item, the effects of all physical parameters resulting from the current study on the distributions of axial velocity, temperature, solutal concentration, nanoparticles volume fraction, pressure gradients, and streamlines profiles will be discussed in detail with the natural or the physical meanings for each parameter when the rest of the parameters are fixed as follows, $\Omega =1, Bn=0.5, a=0.8, {Gr}_{T}={Gr}_{c}={Gr}_{p}=0.5, \rho =2.6, \mu =2.6, Nb=Nt=0.5, Ec=0.6, M=1, {\theta }_{w}=0.6, \beta =-0.1, R=0.8, {N}_{T\mathcal{F}}=0.4, {N}_{\mathcal{F}T}=0.5, {Bi}_{1}={Bi}_{2}=0.4, {Mi}_{1}={Mi}_{2}=0.5, n=3, \eta =0.6$ .

### Axial velocity distribution

**Figure 2:**
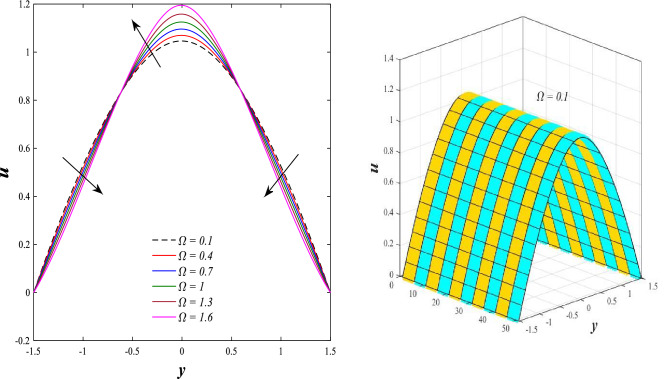
Effect of Rotation Ω on the axial velocity distribution 2D and 3D.

**Figure 3:**
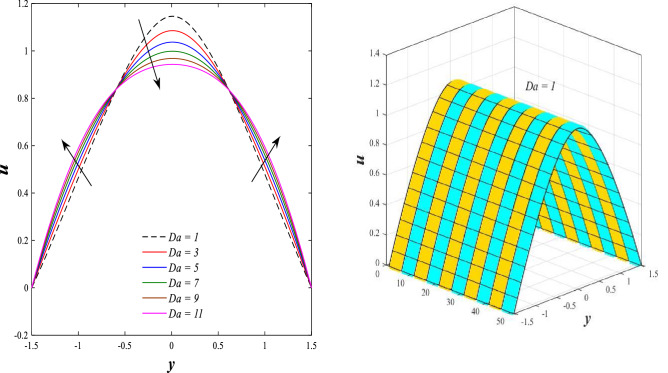
Effect of Darcy number *Da *on the axial velocity distribution 2D and 3D.

**Figure 4:**
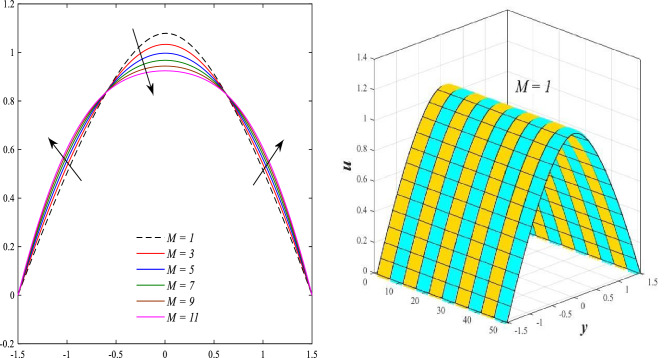
Effect of magnetic field parameter M on the axial velocity distribution 2D and 3D.

**Figure 5:**
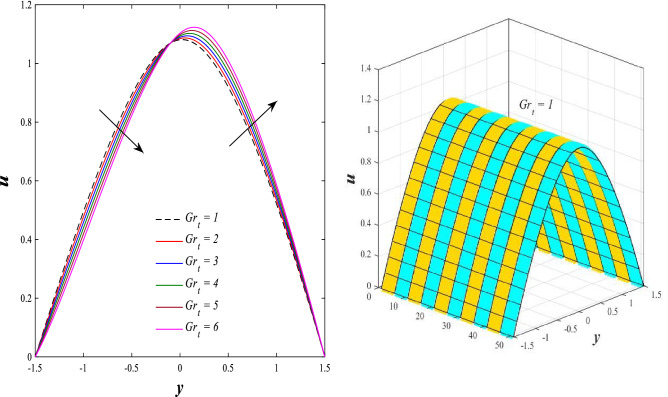
Effect of heat Grashof number *Gr*_*t*_ on the axial velocity distribution 2D and 3D.

**Figure 6:**
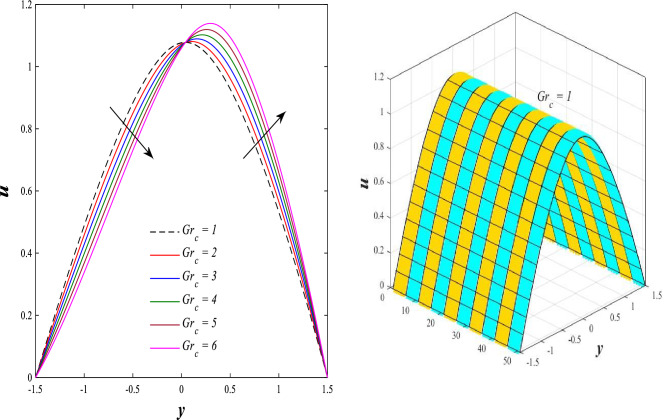
Effect of solutal Grashof number *Gr*_*c*_ on the axial velocity distribution 2D and 3D.

**Figure 7:**
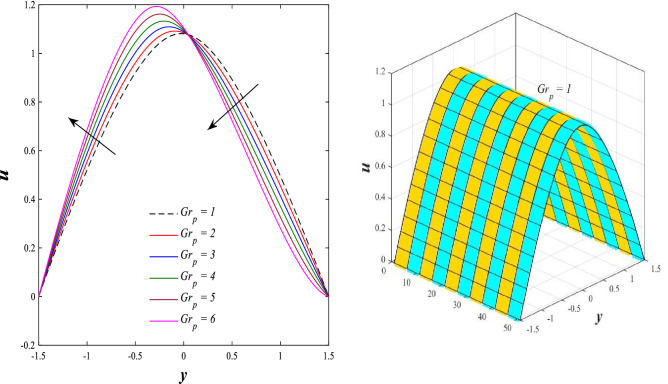
Effect of nanoparticles Grashof number *Gr*_*p*_ on the axial velocity distribution 2D and 3D.

**Figure 8:**
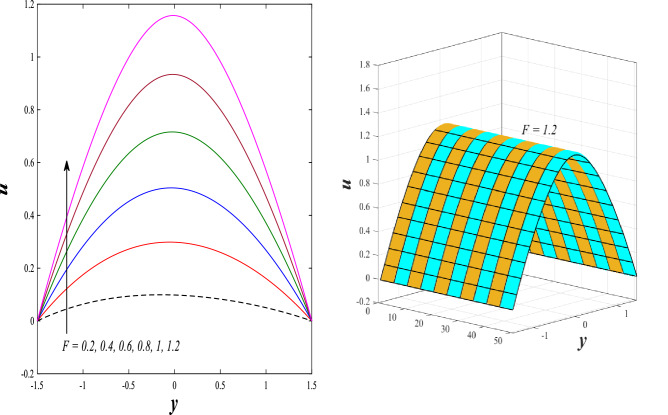
Effect of mean flow rate *F* on the axial velocity distribution 2D and 3D.

Figure 2 illustrates the relationship between rotation $\Omega$ and axial velocity distribution $u$ when the rest of the parameters are constant. It has been observed that the behavior of the axial velocity distribution when boosting rotation changes from decreasing in the range $-1.5\leq y \leq-0.5$ from the beginning of the channel then to increasing in the range $-0.5\leq y \leq0.5$ inside the channel and then decreasing again in range $0.5\leq y \leq1.5$ to the end of the channel, in other words, the increase in the values of the rotation $\Omega$ lead to decrease the axial velocity distribution at the channel walls and increase it at the middle of the channel, and this reflects us the fact that rotation creates an irregular behavior in the axial velocity of the fluid inside the channel. Figures 3 and 4 were plotted to show the behavior of the axial velocity distribution under the influences of the different increasing values of the Darcy number $Da$ and the magnetic field parameter $M$ when the rest of the parameters are constant. It was found that the axial velocity distribution has several varying changes between ups and downs, in the domain $-1.5 \leq y \leq-0.5$ from the bottom wall of the channel, the effect of the two parameters on the axial velocity distribution is positive, while the opposite occurs in the domain $-0.5\leq y \leq0.5$ inside the channel, and finally, the effect becomes positive again in domain $0.5\leq y \leq1.5$ in other words, the enhancement in the values of the Darcy number $Da$ and the magnetic field parameter $M$ lead to enhancement of the axial velocity distribution at the channel walls and reduce it at the middle of the channel. and this is a very noticeable discrepancy for the axial velocity distribution in this case. Physically, the enhancement of the different values taken by Darcy's number and their effect on the axial velocity distribution caused an increase in fluid velocity at the walls of the channel and the reason for this is that the density of the porous medium at the walls of the channel is small, while the axial velocity at the center of the channel or the channel axis is low because the density of the porous medium in these the area is large. On the other hand, it is known that the strength of the magnetic field is a resistive force that hinders the movement of the fluid in general and since the fluid under study flows inside a closed channel with flexible walls the strength of the magnetic field controls the movement of the fluid at the axis of the channel, causing a decrease in the fluid velocity while the movement of the fluid at the walls of the channel is not affected by the strength of the magnetic field so, the axial velocity of the fluid at the walls of the channel becomes increasing. In the end, the porous medium and the magnetic field both represent resistance forces that directly affect the axial velocity distribution, whether at the walls of the flexible channel or the axis of the channel, and the effect of this force appears clearly at the center of the channel, which negatively affects the fluid velocity. Figures 5, 6, and 7 show the effects of the heat Grashof number ${Gr}_{t}$, the solutal Grashof number ${Gr}_{c}$, and finally the nanoparticles Grashof number ${Gr}_{p}$ on the axial velocity distribution $u$. The decrease in axial velocity distribution was observed starting from the wall of the lower channel to the inside of the channel until its middle, in the ambit $-1.5\leq y \leq0,$ and soon the effect turns into growth in the axial velocity distribution from the middle of the channel to the upper wall of the channel in the ambit $0 \leq y \leq1.5$ under the influences each of heat and solutal Grashof numbers, but in the case of the nanoparticles Grashof number the exact opposite occurs, the axial velocity distribution begins with an increase and then ends with a decrease. Physically, It is necessary to note that the ratio between the thermal buoyancy force of the fluid and the viscous hydrodynamic force is a non-fixed ratio that indicates that the increase in the thermal buoyancy force of the fluid directly leads to a weakening of the fluid viscosity, which makes the fluid movement easier and vice versa, based on what was mentioned, the heat Grashof number expresses the relative relationship between the thermal buoyancy force of the fluid and the viscous hydrodynamic force. Accordingly, in the distance $0\leq y \leq1.5$ of the channel, the enhancement values of the heat Grashof number lead to a decrease in the fluid viscosity rate and thus an increase in the fluid velocity distribution while the opposite occurs in the distance $-1.5\leq y \leq0$ of the channel. As for the solutal Grashof number, its behavior is the same as that of the heat Grashof number. In distance $-1.5 \leq y \leq0$ of the channel the axial velocity distribution increases with increasing values of the nanoparticle's Grashof number due to the decrease in fluid viscosity, while in distance $0 \leq y \leq1.5$ of the channel the opposite occurs. Figure 8 shows the effect of enhancing the values of mean flow coefficient $F$ on the fluid axial velocity distribution within the elastic channel. It has been shown that the larger values ​​of the mean flow coefficient, lead to an enhancement in the distribution of fluid velocity. From the point of view of the physical nature, there is an agreement of the positive relationship between the distribution of velocity and the average flow which acts as a force that pushes the fluid into the channel, and thus its velocity increases to a large extent.

### Temperature, Solutal, and nanoparticle volume fraction distributions

**Figure 9:**
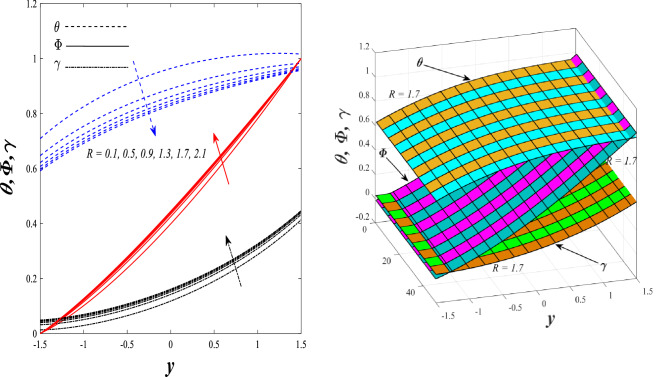
Effect of non-linear thermal radiation parameter *R* on the distributions of both temperature, solutal concentration and nanoparticles volume fraction 2D and 3D.

**Figure 10:**
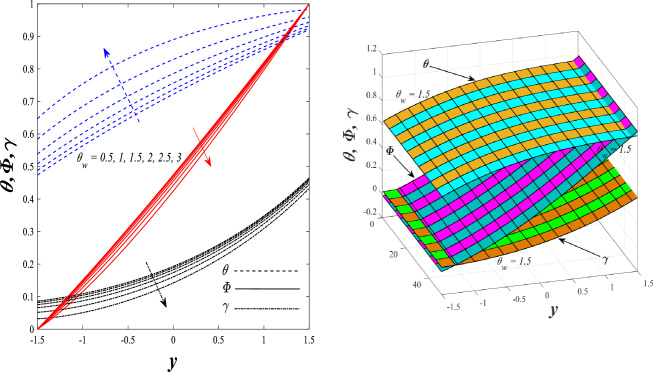
Effect of temperature ratio parameter* θ*_*w*_ on the distributions of both temperature, solutal concentration and nanoparticles volume fraction 2D and 3D.

**Figure 11:**
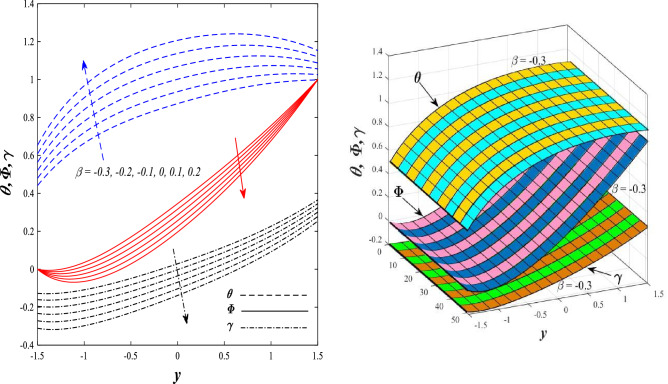
Effect of heat generation/absorption parameter β on the distributions of both temperature, solutal concentration and nanoparticles volume fraction 2D and 3D.

**Figure 12:**
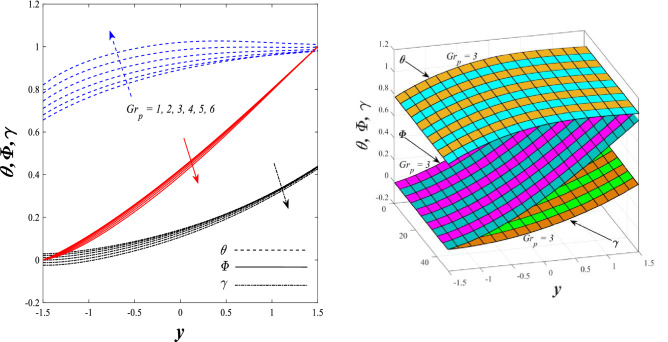
Effect of nanoparticles Grashof number *Gr*_*p*_ on the distributions of both temperature, solutal concentration and nanoparticles volume fraction 2D and 3D.

**Figure 13:**
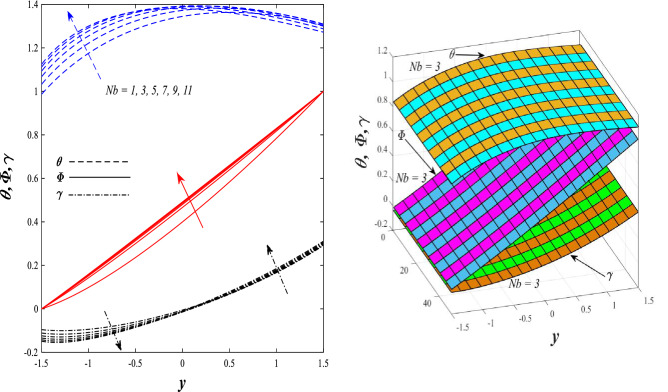
Effect of Brownian motion parameter *Nb* on the distributions of both temperature, solutal concentration and nanoparticles volume fraction 2D and 3D.

**Figure 14:**
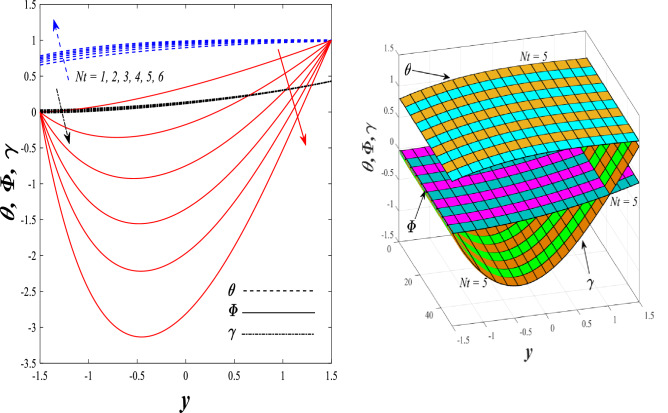
Effect of thermophoresis parameter *Nt* on the distributions of both temperature, solutal concentration and nanoparticles volume fraction 2D and 3D.

**Figure 15:**
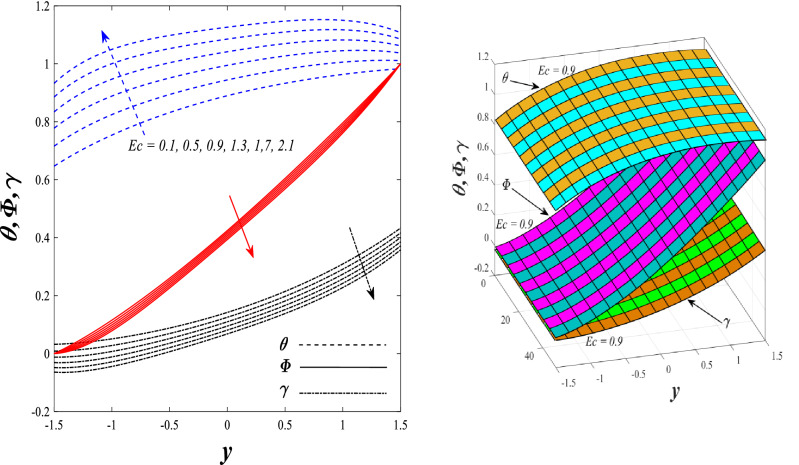
Effect of Eckert number *Ec* on the distributions of both temperature, solutal concentration and nanoparticles volume fraction 2D and 3D.

**Figure 16:**
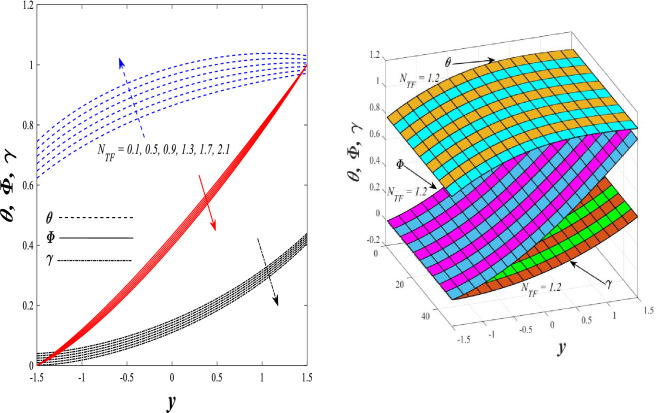
Effect of Dufour parameter *N*_*T*ℱ_ on the distributions of both temperature, solutal concentration and nanoparticles volume fraction 2D and 3D.

**Figure 17:**
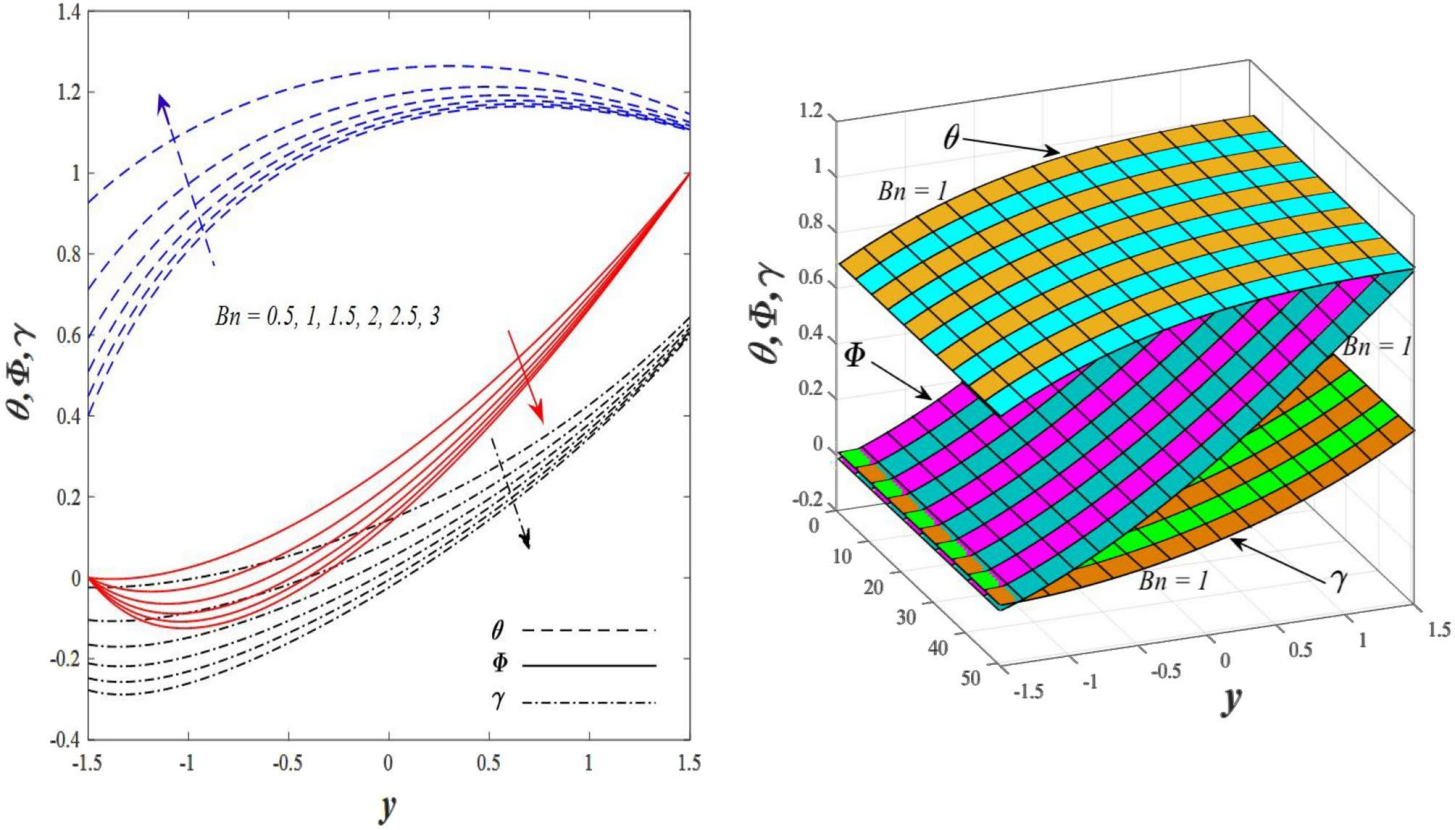
Effect of Brinkman number *Bn* on the distributions of both temperature, solutal concentration and nanoparticles volume fraction.

**Figure 18:**
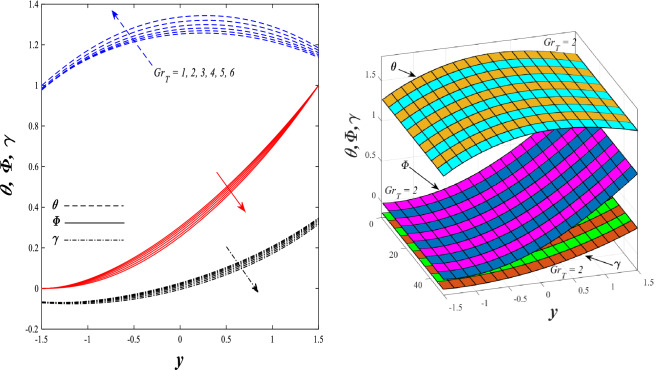
Effect of heat Grashof number *Gr*_*t*_ on the distributions of both temperature, solutal concentration and nanoparticles volume fraction 2D and 3D.

**Figure 19:**
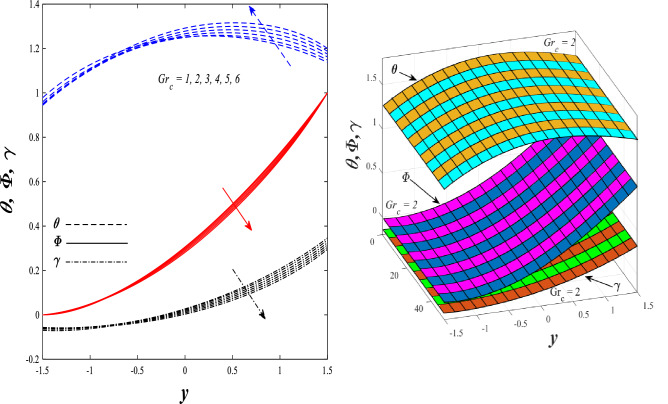
Effect of solutal Grashof number *Gr*_*c*_ on the distributions of both temperature, solutal concentration and nanoparticles volume fraction 2D and 3D.

**Figure 20:**
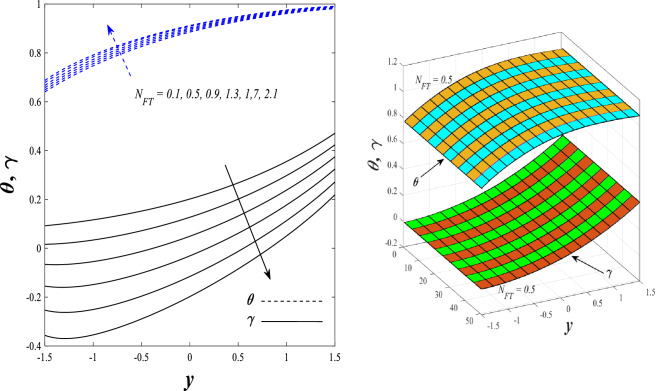
Effect of Soret parameter *N*_ℱ*T*_ on the distributions of both temperature and solutal concentration 2D and 3D.

**Figure 21:**
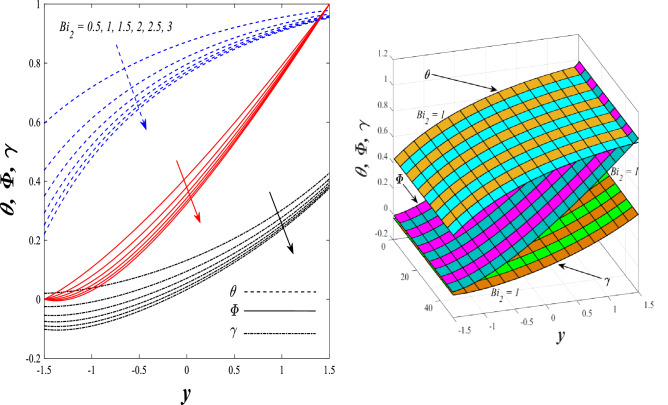
Effect of heat Biot number *Bi*_2_ on the distributions of both temperature, solutal concentration, and nanoparticles volume fraction.

**Figure 22:**
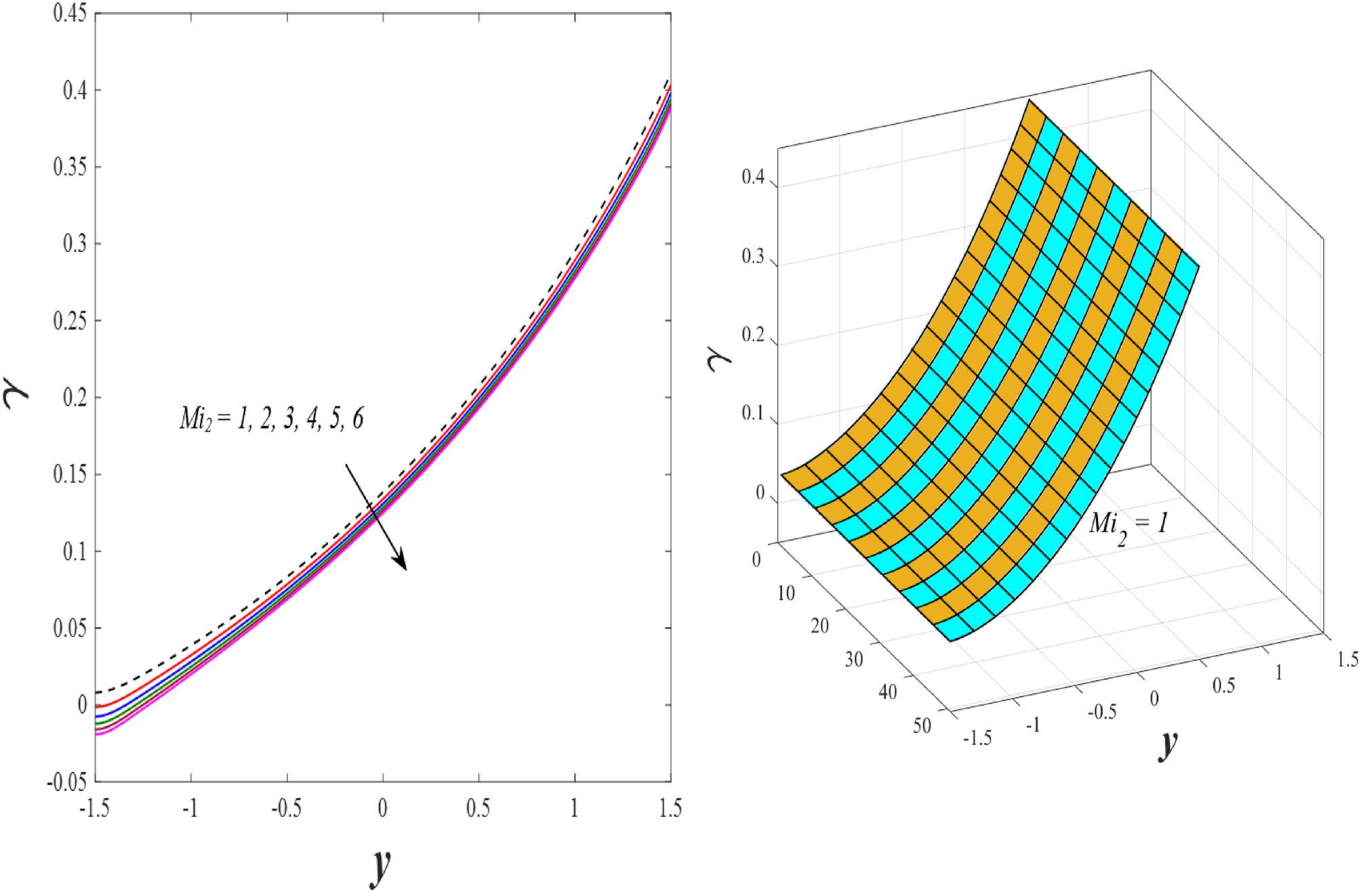
Effect of mass Biot number *Mi*_2_ on the distribution of solutal concentration.

Figures 9 and 10 displays the impact of the nonlinear thermal radiation parameter $R$ and the temperature ratio parameter ${\theta }_{w}$ on the distributions of temperature $\theta$, solutal concentration $\gamma ,$ and nanoparticles volume fraction $\Phi$, It can be seen from these two figures that both parameters negatively affect the temperature distribution and positively affect the solutal concentration distribution and nanoparticles volume fraction distribution. Physically, the thermal conductivity coefficient $k$ controls the mechanism of influence on the nonlinear thermal radiation parameter $R$ and the temperature ratio parameter ${\theta }_{w}$, so that the relationship between the thermal conductivity coefficient $k$ and the previous two parameters is an inverse relationship. Thus, the reason for the decrease in the temperature distribution when the values of the previous two parameters are enhanced is the occurrence of thermal diffusion away from the system due to the thermal conductivity coefficient $k$. In other words, the relationship between thermal diffusion and the coefficient of thermal conductivity became an inverse relationship caused by the effect of the nonlinear thermal radiation parameter and the temperature ratio parameter on the temperature distribution, and the walls of the channel also affect the occurrence of thermal diffusion far from the fluid or the system. As for studying the influences of the heat generation and absorption parameter $\beta$, as well as the effect of the nanoparticles Grashof number ${Gr}_{p}$ on the variations that occur in distributions of temperature $\theta$, solutal concentration $\gamma$, and nanoparticles volume fraction ${\Phi }$, you should, dear reader, look at Figs. 11 and 12. It explains the enhancement in the values of these two parameters is accompanied by an increase in the temperature distribution on the one hand, and a decrease in the solutal distribution and the nanoparticles volume fraction distribution on the other hand. Physically, the increase in the temperature distribution field transport from heat absorption $(\beta <0)$ into heat generation $(\beta >0)$ by the average kinetic energy of the fluid particles, which leads to an increase in the movement and speed of the fluid molecules within the channel because the temperature determines the kinetic energy associated with the movement of the fluid molecules and nanoparticles, which also causes small distances between the fluid molecules. Figure 13 plays an important role in showing the Brownian motion parameter $Nb$ effect on the distributions of temperature $\theta$, solutal concentration $\gamma$, and nanoparticle volume fraction $\Phi$, the increase in its different values caused a growth in the distributions of both temperature and nanoparticle volume fraction with an increase and decrease in the distribution of solute concentration. Physically, the enhancement of the Brownian motion coefficient increases the distributions of temperature, solutal concentration, and nanoparticles volume fraction due to the random movement of the fluid and nanoparticles together, which is controlled by the Brownian motion coefficient, which leads to a direct positive relationship between the random motion system and the previous three major distributions. On the other side, Fig. 14 was plotted specifically to illustrate the change in the behavior of the distributions of both the temperature $\theta$, solutal concentration $\gamma$, and nanoparticles volume fraction $\Phi$ under the influence of the increase in values of the thermophoresis parameter $Nt$. This effect of this parameter caused a significant advance in the temperature distribution $\theta$, and a marked decrease in the other two distributions $\gamma$ and $\Phi$. Physically, the phenomenon of thermophoresis resulting from strengthening values of the thermophoresis parameter causes the migration or transfer of fluid particles and nanoparticles with them from the less heated medium to the hotter medium, causing an increase in the thermal diffusion rate and an increase in the temperature distribution profile and a decrease in the distributions of both solutal concentration and nanoparticle volume fraction. Figure 15 gives a graphic relationship between the Eckert number $Ec$ and the three main distributions, namely temperature $\theta$, solutal concentration $\gamma$, and nanoparticle volume fraction $\Phi$. It was found that the effect of the Eckert number $Ec$ on the temperature distribution $\theta$ is positive, while its effect on the two other distributions $\gamma$ and $\Phi$ is negative. Physically, it is known that the Eckert number is a dimensionless quantity that expresses the kinetic energy and the enthalpy difference of the elastic channel and is used to describe the heat transfer dissipation, when the values ​​of the Eckert number are strengthened, thermal diffusion is generated near the system, which means an increase in the temperature distribution, while the resulting dissipation causes a decrease in the distributions of both the solutal concentration the nanoparticles volume fraction. In Fig. 16, the relationship between $\theta , \gamma ,\Phi ,$ and $y$ is obtained for different values of the Dufour parameter ${N}_{T\mathcal{F}}$. From this figure, the temperature distribution $\theta$ increases with increasing ${N}_{T\mathcal{F}}$, and the distributions of both the solutal concentration $\gamma$ and the nanoparticle volume fraction $\Phi$ decrease against the increase in the values of ${N}_{T\mathcal{F}}$. As for the change in the behavior of the distributions of both temperature $\theta$, solutal concentration $\gamma$, and nanoparticle volume fraction $\Phi$ as a result of affecting them with Brinkman number $Bn$, you can find it, dear reader, in Fig. 17 you may notice a significant enhancement in the temperature distribution and deterioration in the two others distributions when the Brinkman $Bn$ number values are enhanced. Physically, the Brinkmann number is a dimensionless quantity that expresses the process of heat transfer or heat conduction from a wall to inside a viscous flowing fluid. Accordingly, the increase in values of the Brinkmann number means an increase in the thermal conductivity or an increase in the thermal diffusion from the walls of the channel to inside the fluid and thus plays an important role in enhancing the temperature of the fluid. Figures 18 and 19 show the effects of the heat Grashof number ${Gr}_{t}$ and solutal Grashof number ${Gr}_{c}$ on the distributions of temperature $\theta$, solutal concentration $\gamma$, and nanoparticle volume fraction $\Phi$. There is a growth in the temperature distribution and a decline in the distributions of both solutal concentration and nanoparticle volume fraction. Physically, the relative relationship between the thermal buoyant force of the fluid and the viscous hydrodynamic force of the fluid is determined by the heat Grashof number. Accordingly, the higher the heat Grashof number values, the more the thermal buoyancy force overcomes the viscous hydrodynamic force of the fluid, which causes a decrease in viscosity and therefore a rise in the temperature distribution file, which also results in a decrease in the viscosity of nanoparticles inside the fluid, knowing that the solutal Grashof number behaves the same physical path as the heat Grashof number. It is noticeable that the relationship between the temperature distribution $\theta$ and the Soret parameter ${N}_{\mathcal{F}T}$ is a positive direct relationship, in contrast to the relationship between the solutal concentration distribution $\gamma$ and the same parameter, as the enhancement in the values of the Soret parameter leads to an enhancement in the fluid temperature and a decrease in the distribution of solutal concentration see Fig. 20. To know the behavior of the distributions of both temperature $\theta$, solutal concentration $\gamma$ and nanoparticles volume fraction $\Phi$ under influence of the heat Biot number ${Bi}_{2}$, you should, dear reader, look at Fig. 21. You notice that the enhancement in values of the heat Biot number ${Bi}_{2}$ follows a reduction in the previous distributions. Physically, it is known that the heat Biot number is a non-dimensional quantity used in heat transfer calculations, it also helps to know the ratio of thermal resistance inside the fluid and the extent to which it is related to the surface through which the fluid flows (the wall of the elastic channel) so that this ratio works to indicate the different temperatures and in this case the increase in the temperature distribution under the influence of the heat Biot number due to the smallness of the thermal resistance and therefore better thermal diffusion. Finally, Fig. 22 shows the negative effect of the mass Biot number on the distribution of the solutal concentration. It was found that the higher the values of the mass Mass Biot number ${Mi}_{2}$, it led to a slight decrease in the concentration of solute particles, due to the great convergence between the curves of the values of the mass Biot number in this graph.

### Pressure gradients profiles

**Figure 23:**
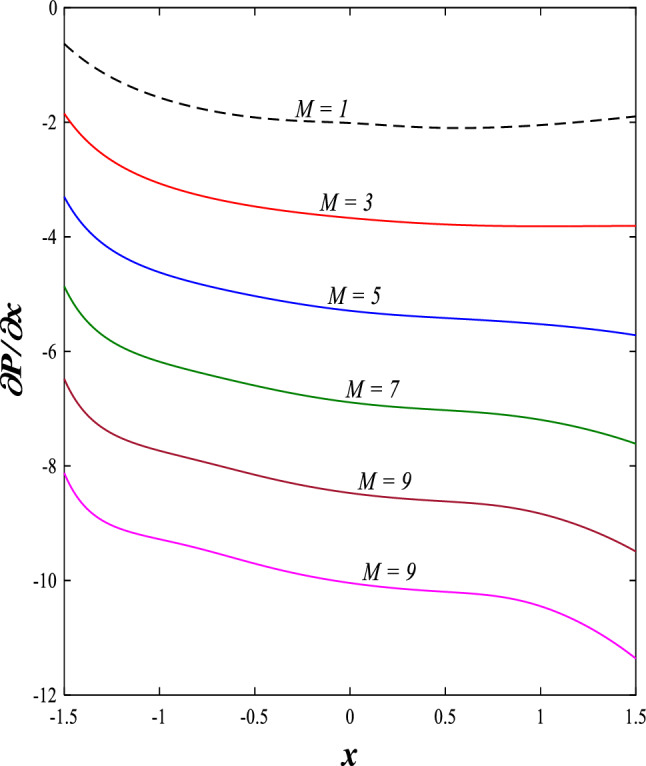
Effect of magnetic field parameter *M* on the pressure gradients distribution.

**Figure 24:**
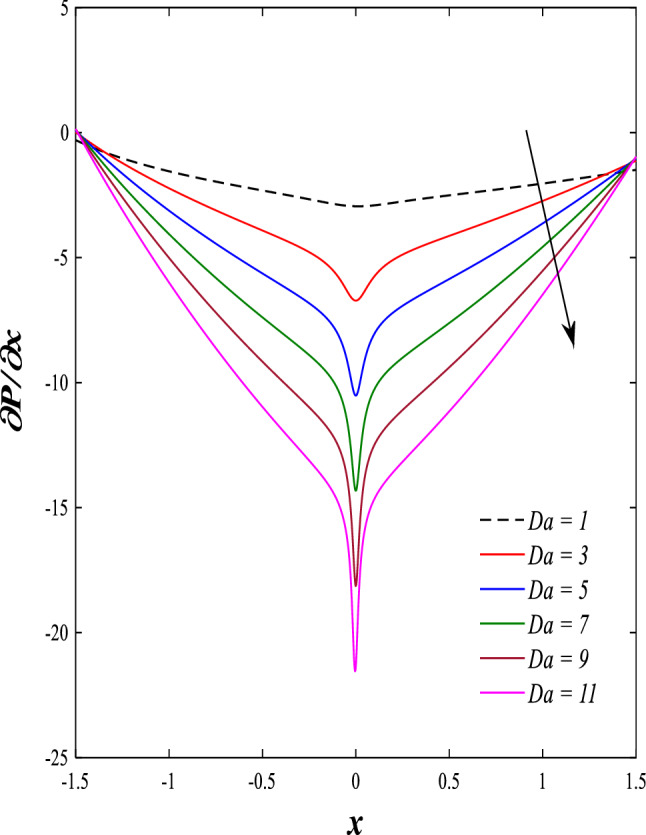
Effect of Darcy number *Da* on the pressure gradients distribution.

**Figure 25:**
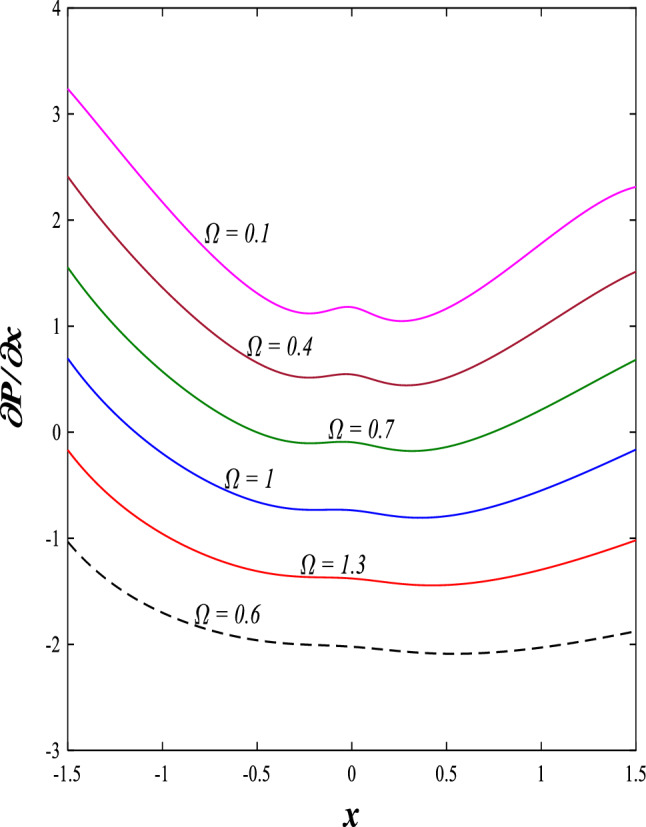
Effect of rotation Ω on the pressure gradients distribution.

**Figure 26:**
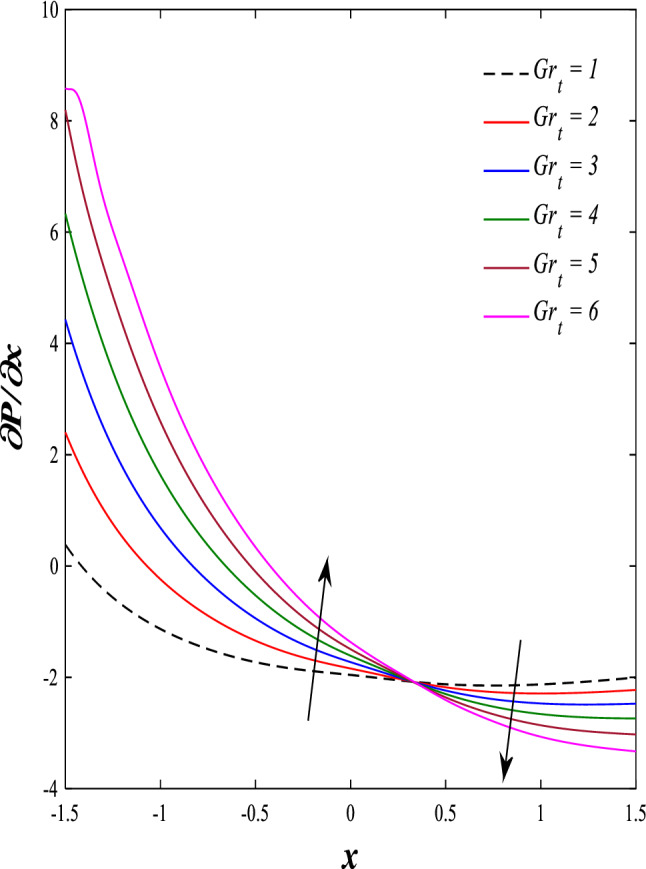
Effect of heat Grashof number *Gr*_t_ on the pressure gradients distribution.

**Figure 27:**
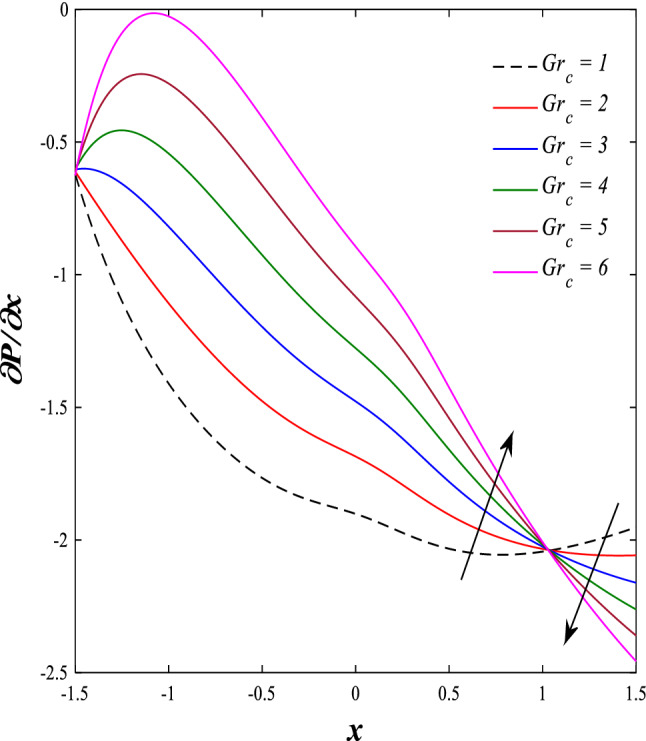
Effect of solutal Grashof number *Gr*_c_ on the pressure gradients distribution.

**Figure 28:**
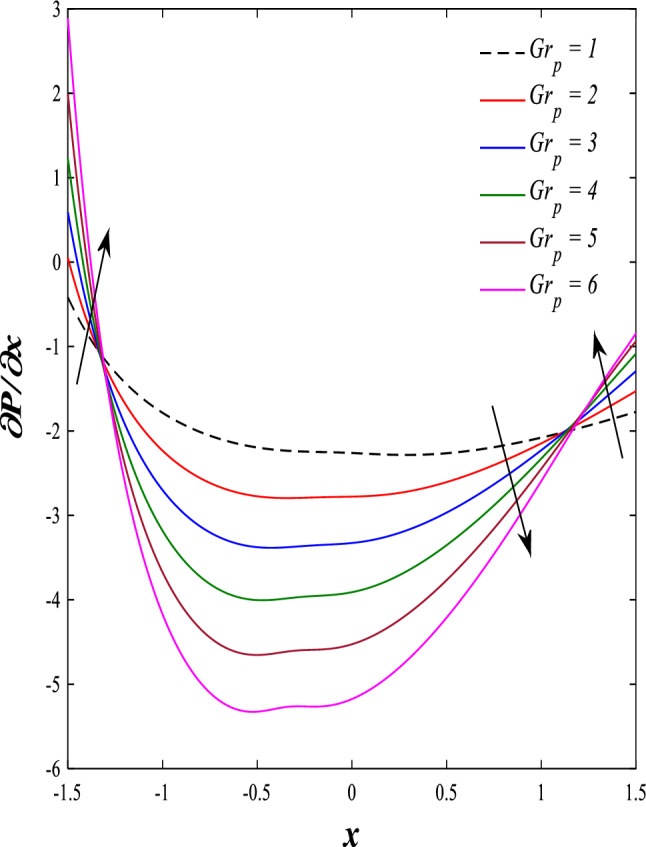
Effect of nanoparticles Grashof number *Gr*_*p*_ on the pressure gradients distribution.

**Figure 29:**
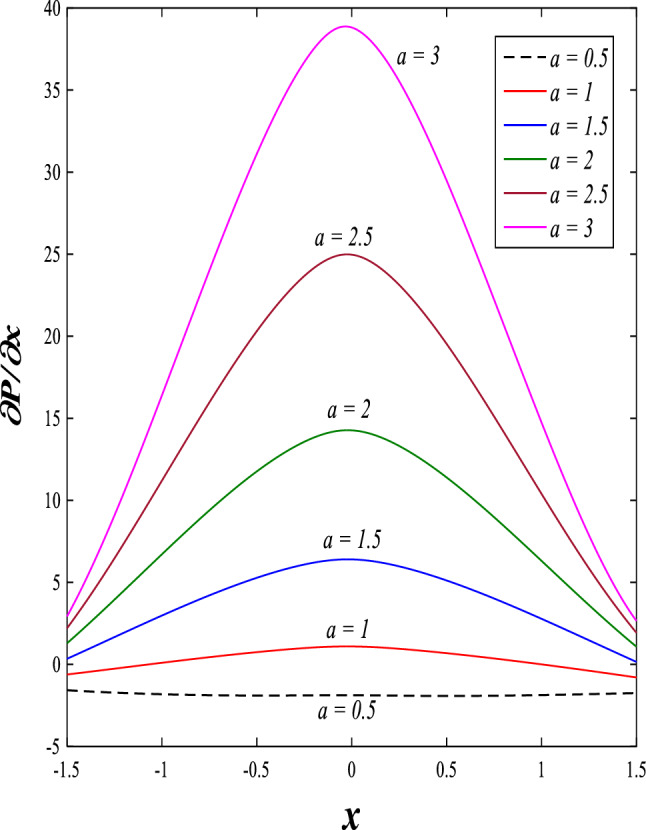
Effect of the half-channel width parameter *a* on the pressure gradients distribution.

It is known that the peristaltic pumping mechanism inside the elastic channels depends entirely on pressure gradients, as it plays an important role in the movement of fluids within these elastic channels. Accordingly, the physical explanations for the effect of some different parameters on the distribution of pressure gradients will be presented as follows; Figs. 23 and 24 show the effect of the magnetic field parameter $M$ and Darcy number $Da$ on the distribution of pressure gradients. It is noticeable that the enhancement in the values of each of them led to a noticeable decrease in the distribution of pressure gradients and this causes a decrease in the fluid's thrust into the channel as a result of the resistance that the fluid meets during its flow. Concerning the effect of rotation $\Omega$ on the distribution of pressure gradients, Fig. 25 shows it. Looking at this figure, it is noted that the relationship between rotation $\Omega$ and the distribution of pressure gradients is a direct positive relationship, meaning that the circulation contributed significantly to the regularity of the fluid movement within the channel while reducing the friction between the fluid and the walls of the channel. Figures 26 and 27 show the behavior of the pressure gradients distribution under the influence of the heat Grashof number ${Gr}_{T}$ and the solutal Grashof number ${Gr}_{c}$. Looking at the previous two figures, you notice dear reader, an irregular behavior of pressure gradients between an increase and a decrease, which causes a disturbance in the pressure gradient distribution. To show the effect of the nanoparticle Grashof number ${Gr}_{p}$ on the distribution of pressure gradients, dear reader, you should look at Fig. 28, and you will find that the effect is irregular between increases and decreases as well, which causes an uneven movement of the fluid inside the channel. The distribution of pressure gradients responds positively when affected by the half-channel width parameter $a$, as the greater the width of the middle of the channel, the more uniformity occurs in the flow force inside the channel resulting from the increase in pressure gradients that is, in general, see Fig. 29.

### Streamlines profiles

**Figure 30:**
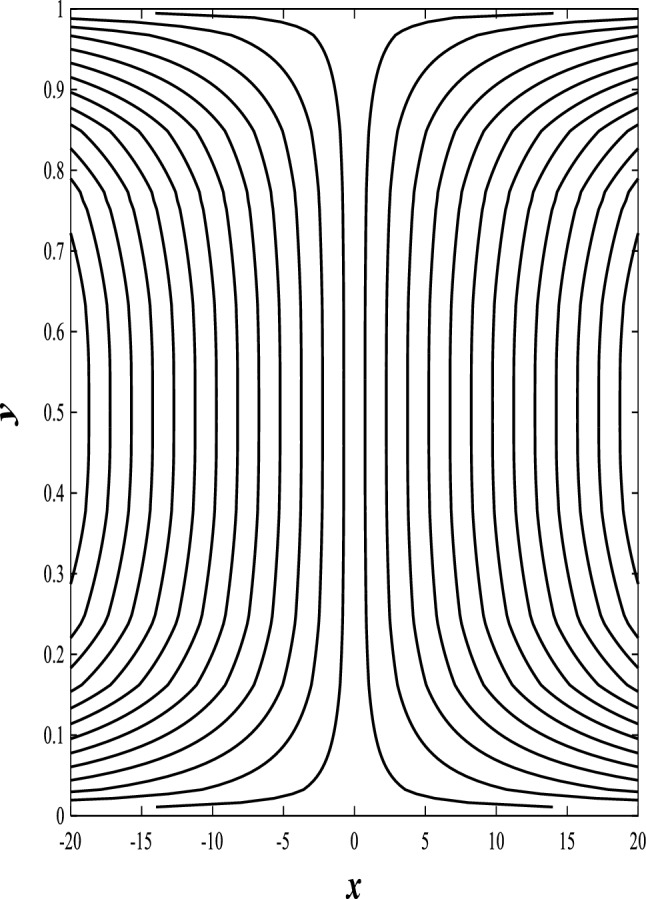
Stream lines profile at *F* = 0.4.

**Figure 31:**
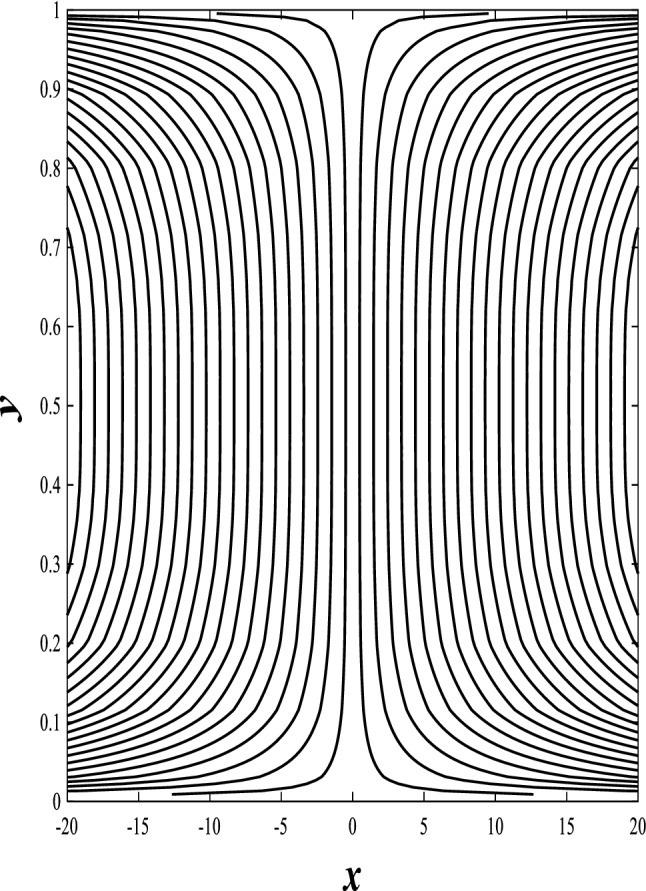
Stream lines profile at *F* = 0.6.

**Figure 32:**
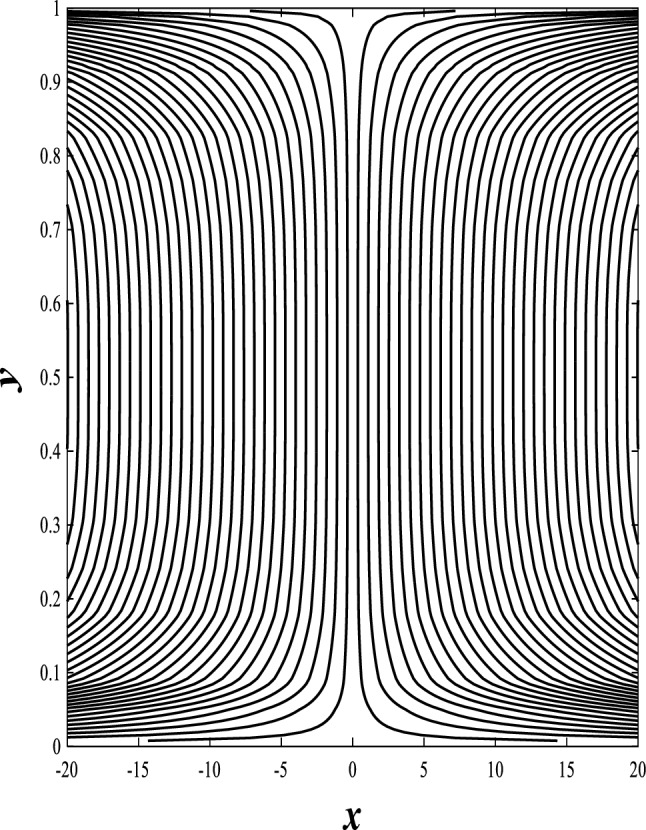
Stream lines profile at *F* = 0.8.

**Figure 33:**
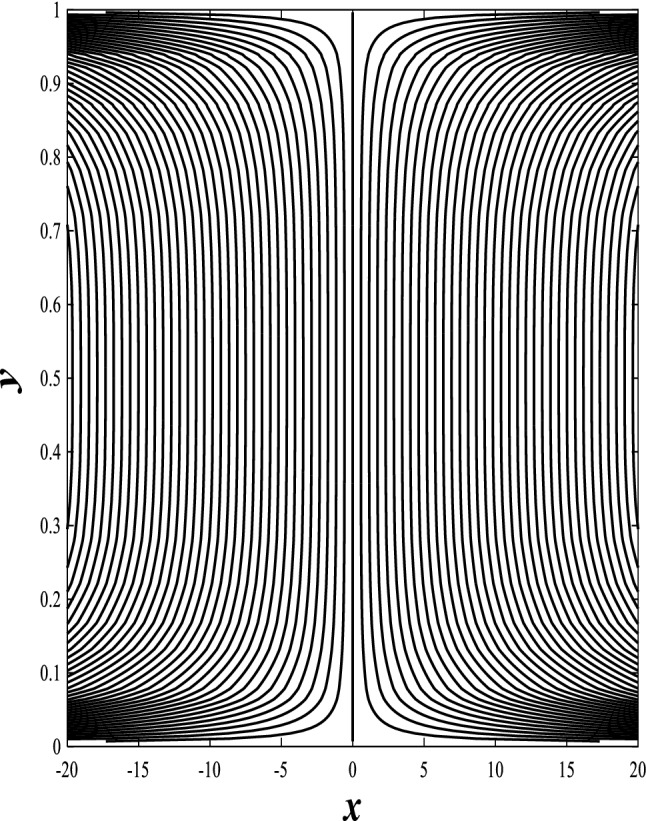
Stream lines profile at *F* = 1.

**Figure 34:**
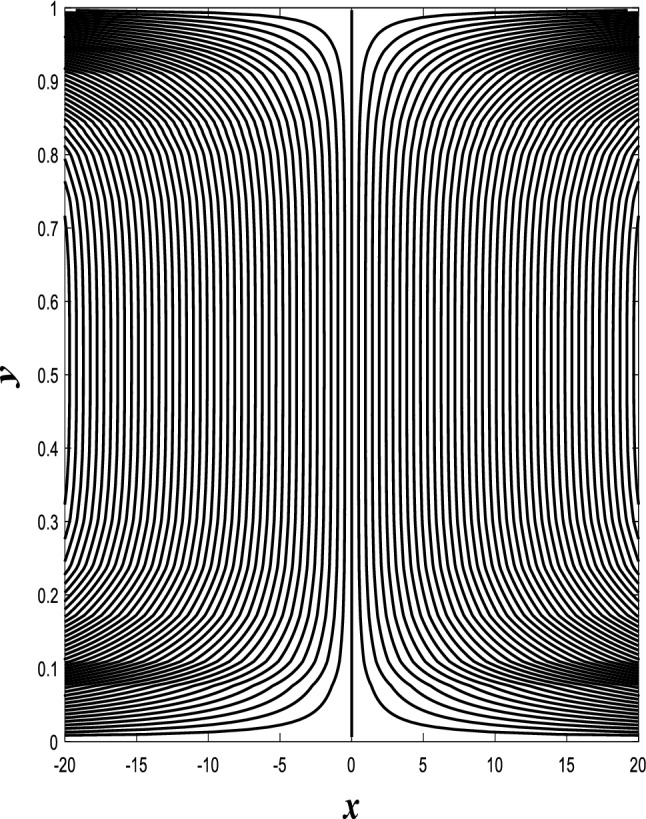
Stream lines profile at *F* = 1.2.

**Figure 35:**
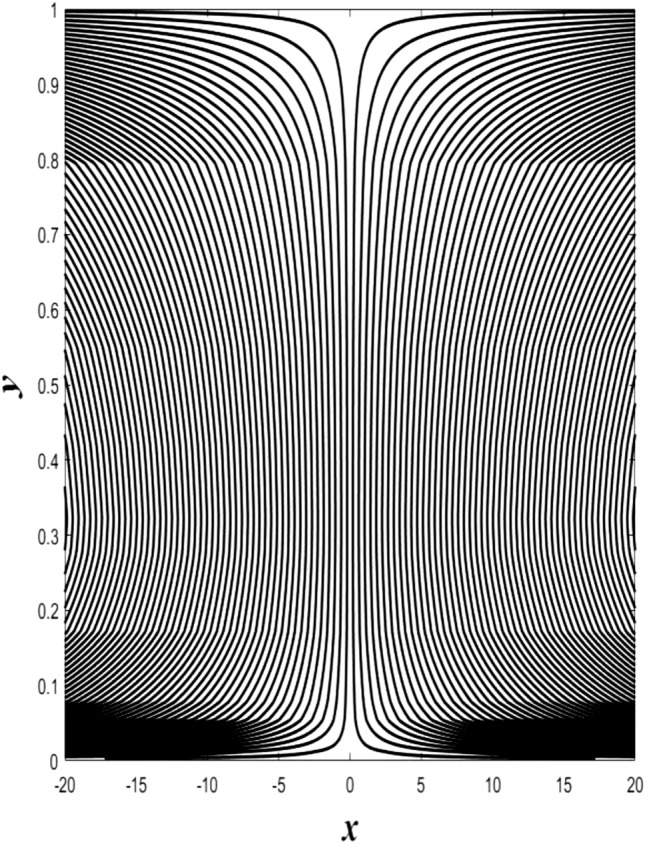
Stream lines profile at *F* = 1.4.

**Figure 36:**
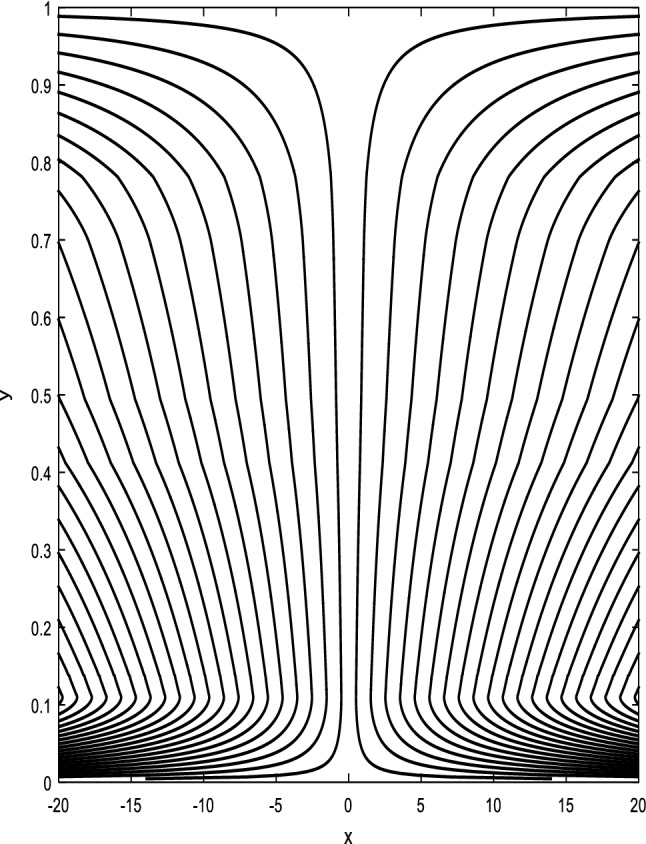
Stream lines profile at *F* = 0.6 and *M* = 1.5.

**Figure 37:**
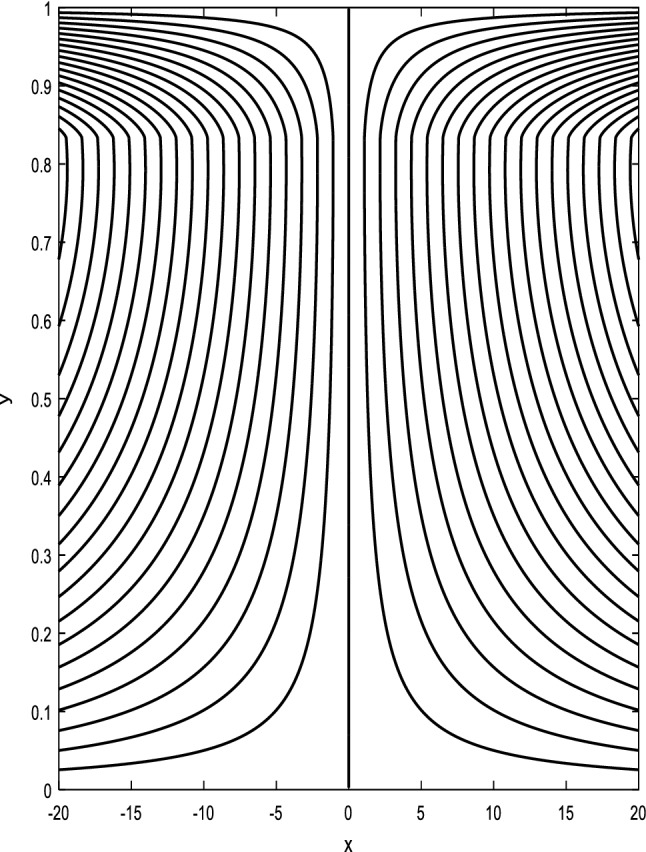
Stream lines profile at *F* = 0.6 and *M* = 2.5.

**Figure 38:**
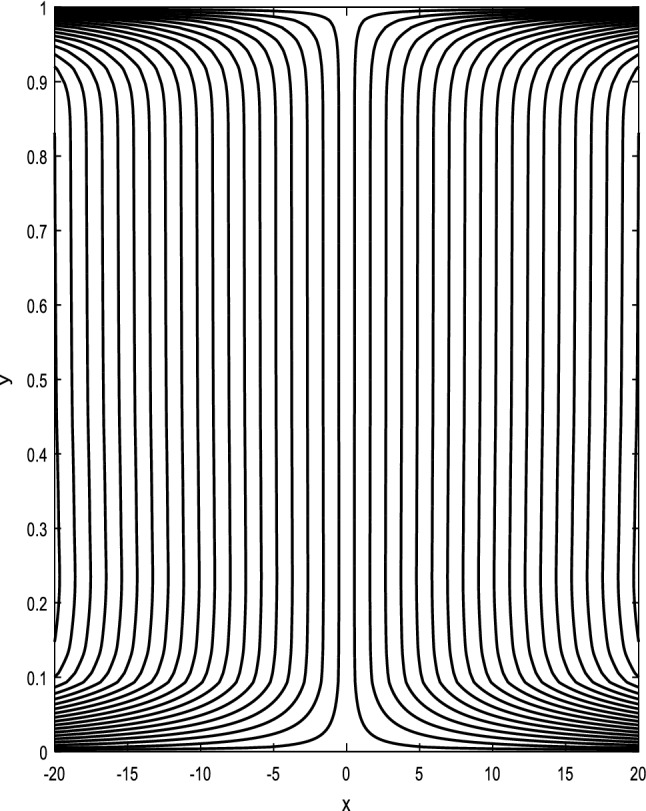
Stream lines profile at *F* = 0.6 and *Da* = 1.

**Figure 39:**
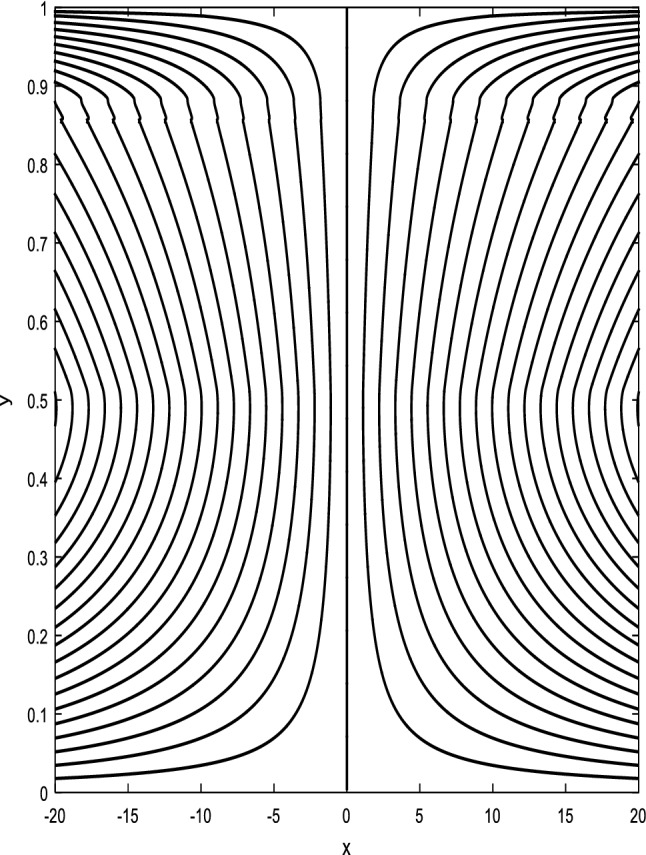
Stream lines profile at *F* = 0.6 and *Da* = 1.5.

**Figure 40:**
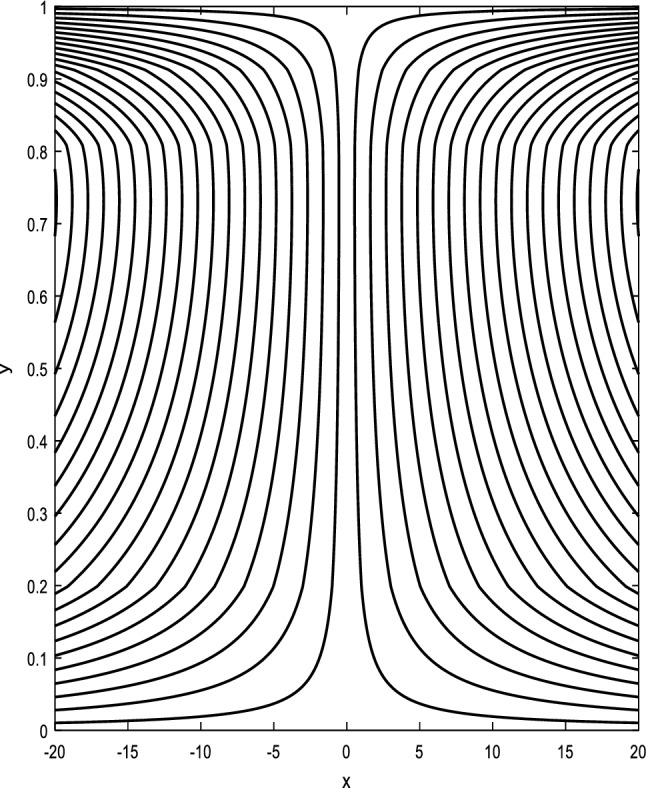
Stream lines profile at *F* = 0.6 and Ω = 0.5.

**Figure 41:**
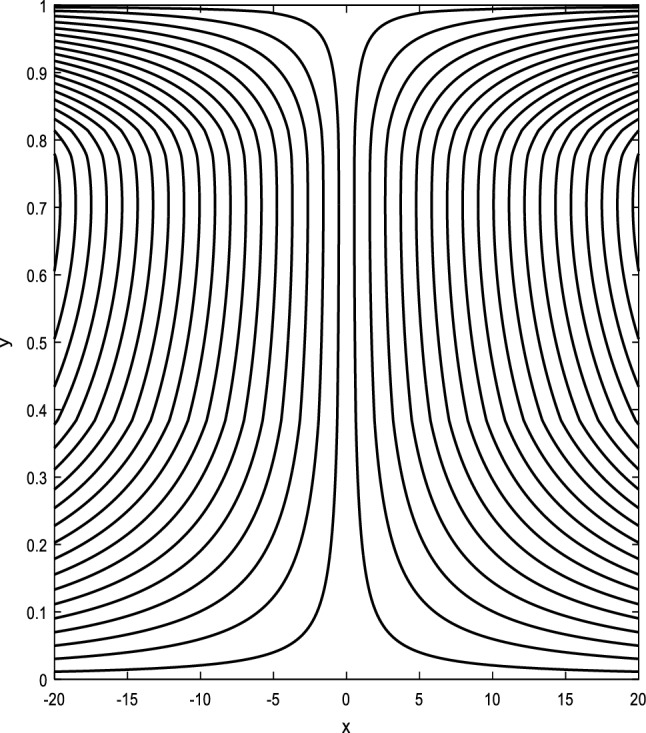
Stream lines profile at *F* = 0.6 and Ω = 1.

**Figures 42:**
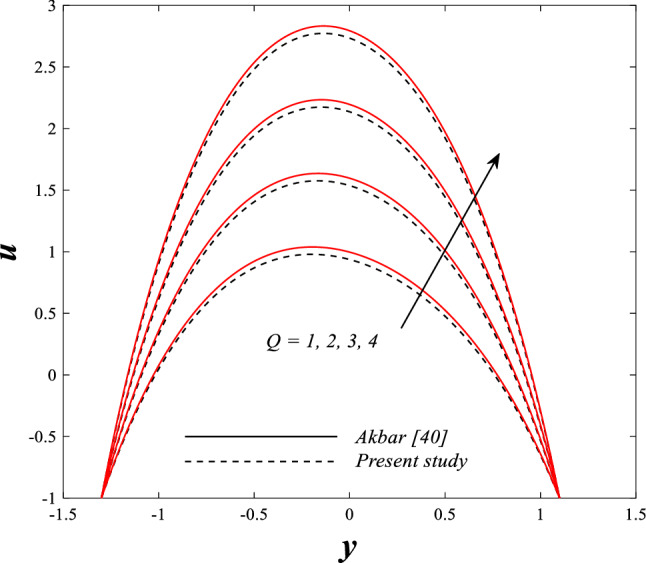
Comparison between Akbar^39^ and present study.

**Figures 43:**
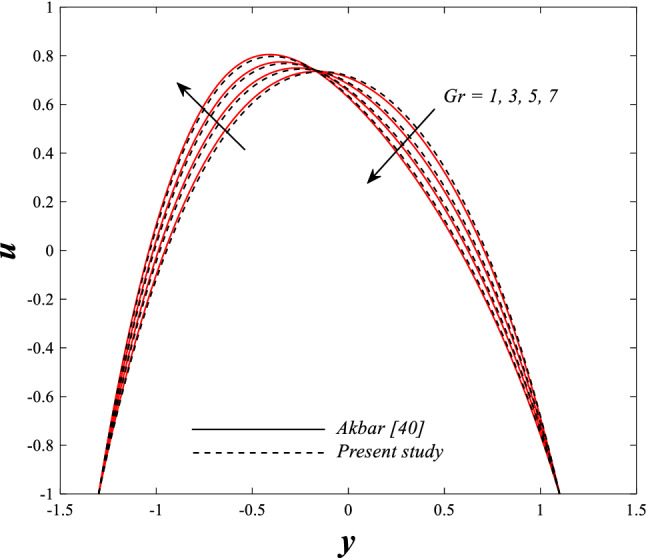
Comparison between Akbar^39^ and present study.

Streamlines play an important role in determining the behavior and characteristics of the flowing fluid in terms of clarifying the field of the flow velocity vector of the fluid for example, through which you can also know whether the flow is regular or irregular (the presence of vortices) and so on. In some detail, the effect of some physical parameters is discussed using graphs and knowing the extent of their influence and their relationship to streamlines as follows; Figs. 30, 31, 32, 33, 34, 35 illustrate the change in the streamlines under the influence of values ​​of the mean flow rate $0.2\le F\le 1$ it was noted that, by increasing the values of the mean flow rate you will find, dear reader, an increase in the streamlines meaning that there is a crowding of the streamlines with the appearance of a bolus on both sides, which indicates an enhancement in the density of the fluid and this is from a physical point of view is normal, as enhancing the mean flow rate leads to an increase in the thrust of the fluid inside the channel. Figures 36 and 37 illustrate the change in the behavior of the streamlines under the influence of two different values of the magnetic field parameter $M$ when the values of the rest of the physical parameters are constant at $F=0.6$. It was found that the convergence in the streamlines at the right side of the channel at $M=1.5$ and that congestion in the streamlines occurs at the lift side of the channel at $M=2.5$ with two boluses occurring right and left the walls of the channel. Figures 38 and 39 show the behavior of the fluid's streamlines during its flow in the channel when the Darcy number takes two different values while the values of the rest of the parameters remain constant at $F=0.6$. The symmetry and regularity of the streamlines on both sides of the channel were observed when $Da = 1$, as well as the occurrence of symmetry of the streamlines around the channel axis with convergence on the right side of the channel when $Da = 1.5$. Finally, Figs. 40 and 41 display the effect of two different values of rotation ${\Omega } = 0.5$ and ${\Omega } = 1$ on the streamlines at $F = 0.6$ and the stability of the rest of the parameters. A slight change was observed in the density and symmetry of the flow lines around the channel axis with the convergence of them on the right side of the channel with the appearance of two boluses on both sides of the channel. It should be noted that when the channel becomes asymmetric and in the absence of the solute concentration equation, porous medium, nonlinear thermal radiation, heat generation/absorption, viscous dissipation, and joule heating, in addition to the absence of heat convection and mass convection from the boundary conditions the current study refers to Akbar ^39^. On the other side, to verify the validity of the numerical method that was used in the current study, a comparison was made using the graphs on the numerical study between the work published by Akbar ^39^ and the present numerical study. Looking at Figs. 42 and 43, show the effect of the parameters $Q$ and $Gr$ on the velocity distribution in the special research study Akbar ^39^ the great convergence between the effect of these parameters on the velocity distribution was observed in the two works together, and this indicates the validity of the numerical method that was used in the current work.

## Conclusion

At the end of this work, it should shed light on the contents of this research, which is the study of the double diffusion of the peristaltic flow process of a non-Newtonian Sisko nanofluid through a suitable porous medium within a symmetric horizontal flexible channel under the influence of the magnetic field, viscous dissipation, heat generation/absorption, and Joule heating in the presence of heat and mass convection taking into account study of the influences of Brownian motion and thermophoresis. On the other hand, the system of partial differential equations governing the flow process has been converted to a system of ordinary differential equations, which has been solved numerically using the Rung–Kutta numerical method with the shooting technique using the code of MATLAB package program. In the end, graphs were made using the MATLAB program which studies the effects of all physical parameters resulting from that important numerical study on the main important distributions, namely axial velocity, temperature, solutal concentration, and finally nanoparticles volume fraction, with clarification of some of the physical meaning of each parameter on one of the previous distributions. Briefly, the results of the current study are presented as follows;

- The axial velocity distribution $u$ is a variable behavior function, its behavior changes between an increase and decreases under the influence of each of the magnetic field parameters $M$, Darcy number $Da$, rotation ${\Omega }$, heat Grashof number $Gr_{T}$, solutal Grashof number $Gr_{c}$, and nanoparticle Grashof number $Gr_{p}$.
- The temperature distribution $\theta$ has become an increasing function under the influence of each of the heat generation/absorption parameters $\beta$, Brinkman number $Bn$, thermophoresis parameter $Nt$, Brownian motion parameter $Nb$, heat Biot number $Bi_{1}$, Dufour parameter $N_{{T{\mathcal{F}}}}$, Eckert number $Ec$, Soret parameter $N_{{{\mathcal{F}}T}}$, heat Grashof number $Gr_{T}$, nanoparticle Grashof number $Gr_{p}$, and solutal Grashof number $Gr_{c}$.
- The temperature distribution $\theta$ decreases when the values of both the nonlinear thermal radiation parameter $R$ and the temperature ratio parameter $\theta_{w}$ are increased.
- The effect of the non-linear thermal radiation parameter $R$, the temperature ratio parameter $\theta_{w}$, and the Brownian motion parameter $Nb$ on the distributions of both the solute concentration $\gamma$, and nanoparticle volume fraction ${\Phi }$ are positive.
- The distribution of the solutal concentration became increasing and decreasing functions under the influence of the heat Biot number $Bi_{1}$ and the soret parameter $N_{{{\mathcal{F}}T}}$, respectively.
- The increase in the values of heat generation/absorption parameter $\beta$, Brinkman number $Bn$, thermophoresis parameter $Nt$, nanoparticle Grashof number $Gr_{p}$, Dufour parameter $N_{{T{\mathcal{F}}}}$, Eckert number $Ec$, heat Grashof number $Gr_{T}$, solutal Grashof number $Gr_{c}$ followed by a decrease in the distributions of both solutal concentration $\gamma$ and nanoparticle volume fraction ${\Phi }$.
- The pressure gradient distribution is a variable behavior function, its wire changes between an increase and decrease under the influence of the heat Grashof number $Gr_{T}$, the nanoparticle Grashof number $Gr_{p}$, and the solutal Grashof number $Gr_{c}$, while this distribution increases with increasing both the rotation ${\Omega }$ and the half-channel width $a$, and this distribution decreases when values of the magnetic field parameter $M$ and Darcy number $Da$ increase.

## Data Availability

The authors declare that all data used in the paper do not use or published elsewhere.

## List of symbols

$\overline{A}_{i}$: The Rivlin–Ericksen tensors
$N_{{T{\mathcal{F}}}}$: The Dufour diffusivity
$a$: The indicates the channel half width
$N_{{{\mathcal{F}}T}}$: The Soret diffusivity
$B_{o}$: The initial magnetic field
$$P$$: The pressure
$Bi_{1}$: The first heat transfer Biot number
$Pr$: The Prandtl number
$Bi_{2}$: The second heat transfer Biot number
$Q_{o}$: The constant of heat conduction
$Bn$: The Brinkman number
$\vec{q}$: The velocity vector
$b$: The wave amplitude
$\vec{q}_{r}$: The radiation heat flux
$\tilde{C}$: The concentration of nanoparticles
$R$: The thermal radiation parameter
$C_{o}$: The initial concentration of nanoparticles
$Re$: The Reynolds number
$c$: The wave speed
$\tilde{S}_{xx} ,\tilde{S}_{xy} ,\tilde{S}_{yy}$: The extra stress tensors
$c_{P}$: The specific heat
$\tilde{T}$: The fluid temperature
$D_{B}$: The Brownian diffusion coefficient
$T_{o}$: The Initial temperature
$D_{T }$: The thermophoresis diffusion
$$tr$$: The trace
$D_{s}$: The solutal diffusivity
$$t$$: The time
$D_{{{\mathcal{F}}T}}$: The Soret diffusivity
$\tilde{U},\tilde{V},\tilde{u},\tilde{v}$: The velocity components in the fixed and wave frames
$D_{{T{\mathcal{F}}}}$: The dufour diffusivity
$\tilde{X},\tilde{Y},\tilde{x},\tilde{y}$: The fixed frame and the wave frame
$D_{m}$: The coefficient of mass diffusivity
$$\lambda$$: The wavelength.
$Ec$: The Eckert number.
$$\sigma$$: Electrical conductivity
$F$: The dimensionless time mean flow rate
$\rho$: The fluid density
$\overrightarrow {{F_{e} }}$: The Lorentz force
$\rho_{p}$: The density of nanoparticles
$Gr_{t}$: The heat Grashof number
$\tilde{\tau }$: The superscript is the matrix transpose
$Gr_{p}$: The nanoparticles Grashof number
$$\Omega$$: The rotation.
$Gr_{c}$: The solutal Grashof number
$\nabla$: The differential operator
$g$: The gravitational acceleration
$\delta$: The wave number
$h^{*}_{1}$: The heat transfer coefficients on the upper wall
$\alpha^{*}$: The material constants
$h^{*}_{2}$: The heat transfer coefficients on the lower wall
$$\beta^{**}$$: The material constants
$\overline{I}$: The identity tensor
$\left( {\rho c_{P} } \right)_{f}$: The heat capacity of the ordinary fluid
$\vec{J}$: The current density
$\left( {\rho c_{P} } \right)_{p}$: The effective heat capacity of the nanoparticles
$J_{he}$: The Joule heating
$\beta$: The heat generation/absorption parameter
$$K_{P}$$: The permeability
$\widetilde{{\mathcal{F}}}$: The solutal (species) concentration
$k^{*}$: The mean absorption coefficient
$\mu$: The dynamic viscosity
$k$: The thermal conductivity
$\psi$: The stream function
$J_{he}$: The Joule heating
${\mathbf{\overline{\rm T}}}$: The Cauchy stress tensor
$L_{1}$: The mass transfer coefficients on the upper wall
$\theta$: The non-dimensional temperature function
$L_{2}$: The mass transfer coefficients on the lower wall
$\beta_{{\mathcal{F}}}$: The volumetric solutal expansion
$M$: The magnetic field parameter
$\beta_{T}$: The thermal expansion coefficient
$Mi_{1}$: The first mass transfer Biot number
$\gamma$: The non-dimensional solutal concentration function
$Mi_{2}$: The second mass transfer Biot number
${\Phi }$: The non-dimensional nanoparticles volume fraction function
$Nb$: The Brownian motion parameter
$Nt$: The thermophoresis parameter

## References

[1] Latham TW (1966). Fluid Motion in a Peristaltic Pump (Master Thesis).

[2] Tanveer A, Hayat T, Alsaed A (2021). Numerical simulation for peristalsis of Sisko nanofluid in a curved channel with double-diffusive convection. Ain Shams Eng. J..

[3] Bibi F, Hayat T, Farooq S, Khan AA, Alsaedi A (2021). Entropy generation analysis in peristaltic motion of Sisko material with variable viscosity and thermal conductivity. J. Therm. Anal. Calorim..

[4] Bhatti MM, Zeeshan A, Ellahi R (2016). Endoscope analysis on peristaltic blood flow of Sisko fluid with titanium magneto-nanoparticles. Comput. Biol. Med..

[5] Almaneea A (2022). Numerical study on the thermal performance of Sisko fluid with hybrid nanostructures. Case Stud. Therm. Eng..

[6] Asghar Z, Ali N, Ahmed R, Waqas M, Khan WA (2019). A mathematical framework for a peristaltic flow analysis of non-Newtonian Sisko fluid in an undulating porous curved channel with heat and mass transfer effects. Comput. Methods Progr. Biomed..

[7] Akram S, Athar M, Saeed K, Razia A (2021). Crossbreed impact of double-diffusivity convection on peristaltic pumping of magneto Sisko nanofluids in non-uniform inclined channel: a bio nanoengineering model. Sci. Prog..

[8] Imran N (2021). the integrated MHD thermal performance with slip conditions on metachronal propulsion the engineering material for biomedical applications. Multidiscip. Model. Mater. Struct..

[9] Sisko AW (1958). The flow of lubricating greases. Ind. Eng. Chem..

[10] Hayat T, Akram J, Zahir H, Alsaed A (2019). Peristaltic motion of Sisko fluid in an inclined asymmetric tapered channel with nonlinear radiation. J. Therm. Anal. Calorim..

[11] Ahmed R, Ali N, Javid K (2019). Heat and mass transfer effects on the peristaltic flow of Sisko fluid in a curved channel. Therm. Sci..

[12] Iqba N, Yasmin H, Kometa BK, Attiya AA (2020). Effects of convection on Sisko fluid with peristalsis in an asymmetric channel. Math. Comput. Appl..

[13] Hayat T, Aslam N, Alsaedi A, Rafiq M (2017). Int. J. Heat Mass Transf..

[14] Nawaz S, Hayat T, Alsaedi A (2020). Numerical study for peristalsis of Sisko nanomaterials with entropy generation. J. Therm. Anal. Calorim..

[15] Imran N, Javed M, Sohail M, Gokul KC, Roy P (2020). Exploration of thermal transport for Sisko fluid model under peristaltic phenomenon. J. Phys. Commun..

[16] Maraj EN, Nadeem S (2015). Theoretical analysis for peristaltic flow of Sisko nanofluid in a curved channel with compliant walls. J. Comput. Theor. Nanosci..

[17] Agoor BM, Ahmed MES, Alam H (2020). Peristaltic flow with heat transfer on Sisko fluid in ciliated arteries. Int. J. Fluid Mech. Thermal Sci..

[18] Rashed ZZ, Ahmed SE (2021). Peristaltic flow of dusty nanofluids in curved channels. Comput. Mater. Continua.

[19] Ahmed SE, Rashed ZZ (2021). Magnetohydrodynamic dusty hybrid nanofluid peristaltic flow in curved channels. Therm. Sci..

[20] Abd-Alla AM, Abo-Dahab SM, El-Seminary RD (2013). Peristaltic flow in cylindrical tubes with an endoscope subjected to the effect of rotation and magnetic field. J. Comput. Theoret. Nanosci..

[21] Abd-Alla AM, Abo-Dahab SM, El-Shahrany HD (2014). Effects of an endoscope and rotation on peristaltic flow in a tube with a long wavelength. J. Comput. Theoret. Nanosci..

[22] Abd-Alla, A. M., Abo-Dahab, S. M., & Albalawi, M. M. Magnetic field and gravity effects on peristaltic transport of a Jeffrey fluid in an asymmetric channel. In *Abstract and Applied Analysis* **2014**, 1–10 (2014).

[23] Abd-Alla AM, Abo-Dahab SM, Kilicman A, El-Semiry RD (2014). Effect of heat and mass transfer and rotation on peristaltic flow through a porous medium with compliant walls. Multidiscip. Model. Mater. Struct..

[24] Mekheimer KS, Abd-Elmaboud Y (2008). Peristaltic flow through a porous medium in an annulus: application of an endoscope. Appl. Math. Inform. Sci,..

[25] Mahmoud SR, Afifi NAS, Al-Isede HM (2011). Effect of the porous medium and magnetic field on peristaltic transport of a Jeffrey fluid. Int. J. Math. Anal..

[26] Tariq H, Khan AA (2020). Peristaltic flow of a dusty electrically conducting fluid through a porous medium in an endoscope. SN Appl. Sci..

[27] Abd-Alla AM, Abo-Dahab SM (2015). Effect of rotation on peristaltic flow of fluid in a symmetric channel through a porous medium with the magnetic field. J. Comput. Theoret. Nanosci..

[28] Abd-Alla AM, Abo-Dahab SM (2015). Magnetic field and rotation effects on peristaltic transport of a Jeffrey fluid in an asymmetric channel. J. Magn. Magn. Mater..

[29] Abd-Alla AM, Abo-Dahab SM, Kilicman A (2015). Peristaltic flow of a Jeffrey fluid under the effect of the radially varying magnetic field in a tube with an endoscope. J. Magn. Magn. Mater..

[30] Choi, S. U. S. Enhancing thermal conductivity of fluids with nanoparticles, in *Proceedings of the ASME International Mechanical Engineering Congress and Exposition*, 99–105 (San Francisco, CA, USA, 1995).

[31] Ahmed SE, Mohamed RA, Aly AM, Soliman MS (2019). Magnetohydrodynamic Maxwell nanofluids flow over a stretching surface through a porous medium: effects of non-linear thermal radiation, convective boundary conditions, and heat generation/absorption. World Acad. Sci. Eng. Technol. Int. J. Aerosp. Mech. Eng..

[32] Mohamed RA, Aly AM, Ahmed SE, Soliman MS (2020). MHD Jeffrey nano fluids flow over a stretching sheet through a porous medium in presence of nonlinear thermal radiation and heat generation/absorption. Transp. Phenom. Nano Micro Scales.

[33] Mohamed RA, Ahmed SE, Aly AM, Chamkha AJ, Soliman MS (2021). MHD Casson nanofluid flow over a stretching surface embedded in a porous medium effect of thermal radiation and slip conditions. Lat. Am. Appl. Res..

[34] Bouslimi J, Omri M, Mohamed RA, Mahmoud KH, Abo-Dahab SM, Soliman MS (2021). MHD Williamson nanofluid flow over a stretching sheet through a porous medium under effects of Joule heating, nonlinear thermal radiation, heat generation/absorption and chemical reaction. Adv. Math. Phys..

[35] Mohamed RA, Abo-Dahab SM, Nofal TA (2010). Thermal radiation and MHD effects on the free convective flow of a polar fluid through a porous medium in the presence of internal heat generation and chemical reaction. Math. Probl. Eng..

[36] Mohamed RA, Ahmed SE, Aly AM, Abo-Dahab SM, Soliman MS (2021). MHD three-dimensional flow of couple stress nanofluids over a stretching sheet through a porous medium in presence of heat generation/absorption and nonlinear thermal radiation. Chall. Nano-Micro Scale Sci. Tech..

[37] Abd-Alla AM, Abo-Dahab SM, El-Shahrany HD (2014). Effects of rotation and initial stress on peristaltic transport of fourth-grade fluid with heat transfer and induced magnetic field. J. Magn. Magn. Mater..

[38] Abd-Alla AM, Mohamed RA, Abo-Dahab SM, Soliman MS (2022). Rotation and initial stress effect on MHD peristaltic flow of reacting radiating fourth-grade nanofluid with viscous dissipation and Joule heating. Waves Random Complex Media.

[39] Akbar NS (2013). Peristaltic Sisko nano fluid in an asymmetric channel. Appl. Nanosci..

[40] Abd-Alla AM, Abo-Dahab SM, El-Shahrany HD (2013). Effect of rotation on peristaltic flow of a micropolar fluid through a porous medium with an external magnetic field. J. Magn. Magn. Mater..

[41] El-Dabe NTM, Moatimid GM, Mohamed MAA, Mohamed YM (2021). A couple stress of peristaltic motion of sutterby micropolar nanofluid inside a symmetric channel with a strong magnetic field and Hall currents effect. Arch. Appl. Mech..

[42] Alhazmi SE, Imran A, Awais M, Abbas M, Alhejaili W, Hamam H, Alhowaity A, Waheed A (2022). Thermal convection in nanofluids for peristaltic flow in a nonuniform channel. Sci. Rep..

[43] Abd-Alla AM, Thabet EN, Bayones FS (2022). Numerical solution for MHD peristaltic transport in an inclined nanofluid symmetric channel with porous medium. Sci. Rep..

[44] Raja MAZ, Sabati M, Parveen N, Awais M, Awan SE, Chaudhary NI, Shoaib M, Alquhayz H (2022). Integrated intelligent computing application for the effectiveness of Au nanoparticles coated over MWCNTs with velocity slip in curved channel peristaltic flow. Sci. Rep..

[45] Hussain S, Raizah Z, Aly AM (2022). Thermal radiation impact on bioconvection flow of nano-enhanced phase change materials and oxytactic microorganisms inside a vertical wavy porous cavity. Int. Commun. Heat Mass Transf..

[46] Hussain S, Aly AM, Alsedias N (2022). Bioconvection of oxytactic microorganisms with nano-encapsulated phase change materials in an omega-shaped porous enclosure. J. Energy Storage.

[47] Hussain S, Alsedias N, Aly AM (2022). Natural convection of a water-based suspension containing nano-encapsulated phase change material in a porous grooved cavity. J. Energy Storage.

[48] Akbar NS, Maraj EN, Noor NFM, Habib MB (2022). Exact solutions of an unsteady thermal conductive pressure driven peristaltic transport with temperature-dependent nanofluid viscosity. Case Stud. Therm. Eng..

[49] Maraj EN, Akbar NS, Iqbal Z, Azhar E (2017). Framing the MHD mixed convective performance of CNTs in rotating vertical channel inspired by thermal deposition: closed form solutions. J. Mol. Liq..

[50] Akram J, Akbar NS, Tripathi D (2022). Electroosmosis augmented MHD peristaltic transport of SWCNTs suspension in aqueous media. J. Therm. Anal. Calorim..

[51] Mohamed RA, Abo-Dahab SM, Soliman MS (2022). Effects of nonlinear thermal radiation and heat generation/absorption on magnetohydrodynamic (MHD) Carreau nanofluid flow on a nonlinear stretching surface through a porous medium. J. Nanofluids.

[52] Abd-Alla AM, Abo-Dahab SM, Abdelhafez M, Thabet EN (2022). Heat and mass transfer for MHD peristaltic flow in a micropolar nanofluid: mathematical model with thermophysical features. Sci. Rep..

